# MMU-STCNN-BDQ: a deep reinforcement learning framework for secure and energy-efficient beamforming in 6G mMIMO networks

**DOI:** 10.1038/s41598-025-26572-2

**Published:** 2026-04-02

**Authors:** Kama Ramudu, Sivasubramanyam Medasani, Tathababu Addepalli, Manish Sharma, Ashish Pandey, Manumula Srinubabu

**Affiliations:** 1 Department of Electronics and Communication Engineering, Aditya University, Surampalem, Andhra Pradesh India; 2 Department of CSE, K. S. School of Engineering and Management, Bengaluru, India; 3 Department of ECE, Aditya University, Surampalem, India; 4 Chitkara University Institute of Engineering and Technology, Chitkara University, Punjab, India; 5 Department of Data Science and Engineering, Manipal University Jaipur, Jaipur, 303007 Rajasthan India

**Keywords:** 6G network, Beam forming, Massive MIMO, Massive Multi-User Spatiotemporal Convolutional Neural Network, Bi-Gated Deep Q-Learning (BDQ), Engineering, Mathematics and computing

## Abstract

To achieve energy-efficient and secure beamforming in 6G massive MIMO networks, especially in millimeter-wave (mmWave) bands. This research work introduces a hybrid framework utilizing the Massive Multi-User Spatiotemporal Convolutional Neural Network (MMU-STCNN) and Bi-Gated Deep Q-Learning (BDQ). The MMU-STCNN extracts secure beamforming prediction vectors from channel state information (CSI) using spatiotemporal and pooling layers enhanced by Multi-User Attention (MUA) mechanisms. These predicted vectors are further optimized through a dynamic bi-gated deep Q-reinforcement learning (BDQ) to enhance both security and energy efficiency. The proposed hybrid architecture addresses the critical challenges in energy efficiency and adversarial robustness, outperforming baseline beamforming methods such as Deep Neural Network (DNN), Reinforcement Deep-Q-Network (DQN), and Adversarial Robust Beamforming (ARBF) across multiple metrics. Experimental results show that the proposed method outperforms DNN, DQN, and ARBF. The proposed method reduces MSE, BER, and increased throughput for 10, 20, and 50 users at SNR levels from 10 to 20 dB. This method is effective for large-scale multi-user communication environments due to its high spectral and energy efficiency. This framework is the key solution for future deployments in 6G mMIMO networks in terms of intelligence, security, and energy efficiency.

## Introduction

The limited spectral resources available for wireless communications are under a lot of strain due to the growing demand for higher data rates. In the 30–300 GHz frequency range, millimeter-wave (mmWave) communication has become a viable option for sixth-generation (6G) and beyond-fifth-generation (B5G) mobile networks. In recent years, mmWave technology has garnered significant research interest due to its broad and underutilised spectrum^1^. mmWave transmission has significant path loss (PL) despite its enormous potential. Massive multiple-input multiple-output (mMIMO) systems can be deployed more easily thanks to the shorter wavelength at mmWave frequencies, which enables the integration of large-scale antenna arrays into small devices. These systems are able to reduce the impact of path loss and substantially increase the signal-to-noise ratio (SNR) because they use spatial beamforming techniques^2^.

Even with advances in digital beamforming technology, large antenna arrays still require separate radio frequency (RF) chains for each antenna element. This limitation results in prohibitively high hardware costs, higher energy consumption, and more complex systems. Conversely, fully analogue beamforming offers limited spectral efficiency and usually supports only one data stream through a shared RF chain, despite being more cost-effective and energy-efficient due to the use of inexpensive phase shifters (PSs)^3^. An approach that aims to alleviate this trade-off by balancing hardware complexity and performance is hybrid analog/digital beamforming. By using more antennas but fewer RF chains, hybrid beamforming finds the best balance between improving signal quality, saving energy, and using spectrum and heat efficiently. However, mmWave signals are best suited for line-of-sight (LoS) communication scenarios because of their vulnerability to interference and signal blockage^4^. Even sophisticated beamforming techniques might not be enough to preserve signal integrity in real-world deployments where LoS paths are frequently blocked, which would significantly reduce the quality of the signal at the receiver.

The rapid proliferation of device-to-device (D2D) communication technologies, such as the Internet of Things (IoT), Industrial IoT (IIoT), and the Internet of Skills (IoS), is expected to make current fifth-generation (5G) wireless networks insufficient in terms of capacity and capabilities. To meet the new demands of the 2030 era, sixth-generation (6G) wireless networks are expected to use terahertz (THz) frequency bands ranging from 0.1 to 10 THz to deliver ultra-high data rates in the terabit-per-second (Tbps) range. To achieve peak capacity and performance, 6G cellular networks must incorporate advanced technologies such as carrier aggregation, ultra-massive MIMO (ultra-mMIMO), cognitive radio, THz communications, and femto cell-based heterogeneous network topologies^5^. Given the complexities of 6G network infrastructure, machine learning (ML) presents a promising data-driven approach for autonomous system operation. Machine learning’s learning, reasoning, and intelligent decision-making capabilities allow networks to dynamically optimise and adapt without human intervention. But adding machine learning to 6G creates new security issues that exacerbate the vulnerabilities already present in wireless communications. A new and significant attack surface in 6G networks is presented by ML-specific vulnerabilities, even though some of the security solutions created for 5G may be modified for AI-driven systems. As a result, creating strong and safe machine learning models is crucial to guaranteeing reliable AI integration in next-generation wireless systems. Prior to the implementation of ML-driven architectures in practical applications, researchers and industry stakeholders need to address these vulnerabilities. To protect 6G network infrastructures from changing threats, it is essential to recognise, record, and assess these new classes of security risks^6^.

Beamforming becomes a crucial enabler as sixth-generation (6G) wireless networks progress towards enabling ultra-reliable, low-latency, and high-throughput communication in dense and heterogeneous environments, particularly in massive MIMO (mMIMO) and millimeter-wave (mmWave) systems. By enabling directional signal transmission, beamforming raises link quality, lowers interference, and increases spectral efficiency. However, beamforming techniques need to be both clever and safe in order to fully take advantage of these advantages in dynamic 6G environments. Machine learning (ML) and deep learning (DL)-powered intelligent beamforming allows for real-time adaptation to changing network topology, user mobility, and channel conditions. This leads to improvements in resource utilisation, energy efficiency, and quality of service (QoS). By incorporating learning-based models, particularly spatiotemporal neural networks and reinforcement learning, systems can predict optimal beam directions and power allocations proactively rather than reactively. These intelligent systems are vulnerable to security threats like model manipulation, adversarial machine learning attacks, and spoofing, though, because they rely on data to make decisions. Thus, secure beamforming is required to protect the availability, confidentiality, and integrity of wireless communication. Critical applications like driverless cars, industrial automation, and remote surgery may suffer from poor performance, user tracking, or even service denial if these mechanisms are not secured. To put it simply, the full potential of 6G depends on the integration of security and intelligence in beamforming. Next-generation wireless systems are reliable because they ensure both optimal performance under complex network conditions and resilience against malicious threats^7^.

The rapid evolution of 6G wireless networks, in particular, requires intelligent, high-throughput, and secures communication solutions for beamforming in massive MIMO (mMIMO) systems operating in the millimeter-wave (mmWave) spectrum. This study proposes a novel architecture called MMU-STCNN-BDQ (Massive Multi-User Spatiotemporal Convolutional Neural Network with Bi-Gated Deep Q-Learning) to get around these challenges. The MMU-STCNN-BDQ framework integrates deep learning and reinforcement learning to generate secure and accurate beamforming predictions. While the spatiotemporal CNN component captures the channel’s temporal and spatial characteristics, the Bi-Gated Deep Q-learning mechanism dynamically optimises beamforming decisions to maximise energy efficiency and minimise interference. This hybrid model effectively balances performance, resource consumption, and security.

This research paper takes a systematic approach to addressing the growing need for secure and intelligent beamforming in 6G wireless networks. Section “Introduction” is an introduction, followed by a detailed Related Work in section “Related works” that discusses recent advances in beamforming, deep learning, and reinforcement learning for wireless communications, as well as scalability, security, and energy efficiency issues. Section “System model and problem formulation” discusses architectural assumptions, channel models, and vulnerabilities in deep learning-based beamforming predictions in dynamic 6G environments. The Proposed Methodology and its discussion in section “Implementation of the proposed methodology” go over the architectural design of the spatiotemporal CNN, the role of bi-gated Q-learning in optimising energy and spectral efficiency, and the hybrid beamforming process, which balances complexity and performance. Section “Experimental setup and its discussion” covers the experimental setup, simulation environment, parameters, and evaluation metrics such as MSE, BER, throughput, and spectral efficiency. The proposed MMU-STCNN-BDQ model is thoroughly examined and compared to conventional methods, demonstrating superior efficiency and robustness under a variety of network conditions and security threats. A security analysis examines machine learning vulnerabilities, adversarial risks, and the framework’s built-in defence strategies. Section “Conclusion and future scope” concludes with a Summary and Future Work, emphasising the significance of secure and intelligent beamforming for 6G networks and proposing future research directions, such as real-world deployment and advanced privacy-preserving learning models. The key novelties of the proposed methodology are summarized as follows

- Proposes a hybrid MMU-STCNN-BDQ framework integrating spatiotemporal CNN and reinforcement learning for adaptive, secure beamforming.
- Introduces a Bi-Gated Deep Q-Learning architecture specifically designed to stabilize learning and improve performance in high-dimensional action spaces.
- Showcases beamforming optimisation with security restrictions in 6G mmWave mMIMO networks for the first time using a combination of discrete and continuous action spaces.
- Offers thorough security analysis and performance comparisons with cutting-edge techniques.

## Related works

### Overview of beamforming in 5G/6G networks

A key component of both 5G and the soon-to-be 6G wireless communication systems, beamforming allows concentrated signal beams to be transmitted in the directions of the intended user, improving signal quality and reducing interference. Beamforming in 5G networks is mostly accomplished by combining massive MIMO systems operating in the sub-6 GHz and mmWave bands with hybrid analog/digital techniques. Higher spectral efficiency, increased coverage, and enhanced capacity are the goals of these systems. The need for ultra-high data rates, ultra-reliable low-latency communication (URLLC), and massive connectivity is growing as 6G is implemented. With the combination of intelligent reflecting surfaces (IRS), terahertz (THz) communication, and AI-driven adaptive algorithms, beamforming in 6G is anticipated to advance even further. 6G beamforming, in contrast to conventional static beamforming techniques, will mainly rely on real-time learning and prediction in order to adjust to extremely dynamic environments and user mobility. Furthermore, the versatility and energy efficiency of beamforming systems are greatly increased by the application of sophisticated machine learning (ML) and deep learning (DL) models in beam selection and prediction. However, issues like hardware complexity, security flaws, and high path loss in mmWave/THz frequencies are still being researched. Therefore, beamforming is a field that is experiencing rapid innovation due to AI, reconfigurable hardware, and intelligent network design, in addition to being a fundamental enabler of 5G/6G performance^8^. Table 1 compares and summarises the different beamforming techniques used in 5G/6G networks based on the literature review. Their main traits, benefits, drawbacks, and suitability for various deployment scenarios are highlighted in this comparison.

**Table 1:** Comparison table of beamforming types.

Beamforming type	Architecture	Advantages	Limitations	Use in 5G/6G
Analog	Single RF chain + Phase shifters	Low hardware cost and power consumption	Limited flexibility; supports one beam	mmWave systems (initial 5G)
Digital	Dedicated RF chains per antenna	High flexibility; supports multi-user MIMO	High cost and power requirements	Sub-6 GHz MIMO, centralized 6G
Hybrid	Few RF chains + phase shifters	Trade-off between cost and performance	Design complexity	Most popular for mmWave 5G/6G
AI-Driven	ML models (DL, RL, etc.)	Adaptive, context-aware, robust to dynamics	Requires training data and computation	Future 6G intelligent networks

### Deep learning (DL) and reinforcement learning (RL) in wireless communication

The development of next-generation wireless systems, such as 5G, 6G, and the Internet of Connected Vehicles (IoCV), is based on the integration of artificial intelligence (AI) and advanced communication technologies^9,10^. More research shows how AI-driven models and modern wireless infrastructures work together, especially to streamline beamforming, channel estimation, and user association. In high SNR environments, extensive beam search through all beam pairs in predefined codebooks yields the best results. However, there is a significant overhead and latency associated with this method. Although hierarchical search techniques are simpler, they frequently fall short in providing coverage at cell borders. Context information (CI)-based beam alignment techniques have been created to address these issues, utilising user-centric parameters like position and orientation to improve the effectiveness of beam selection^11^. The interdependence between localisation and beam management is further reinforced by^12^, which proposed a method that dynamically estimates the receiver’s position and orientation during each step of beam alignment. In^13^, a Bayesian decision-theoretic approach was proposed to address incomplete or uncertain positional data. Effective channel estimation techniques were the focus of early efforts in^14^ with regard to wideband mmWave massive MIMO systems. While^15^ suggested a greedy search-based hybrid beamforming (GS-HB) algorithm assuming perfect channel state information (CSI),^16^ introduced hybrid beamforming for frequency-selective channels using OFDM. In addition,^17^ used the same CSI premise to develop a phase extraction strategy for hybrid precoding. Second-order spatial channel covariance matrices were used in^18^ to further innovate the design of unified analogue beamformers for wideband applications.

Intelligent resource management solutions have been made possible by the incorporation of machine learning (ML) into wireless systems. A framework based on federated learning (FL) was presented in^19^ for the decentralised, real-time optimisation of transmission power allocation and user scheduling in massive MIMO systems. In order to improve user placement, supervised learning (SL) techniques have also been used, outperforming more traditional approaches like support vector machines (SVMs) and k-nearest neighbours (k-NN)^20^. In^21^, a deep learning (DL)-based approach that demonstrated notable gains in complexity and performance trade-offs was put forth to lessen the computational load related to beamforming weight estimation. In order to optimise energy efficiency (EE), hybrid beamforming techniques were investigated in^22^. To solve the sum-rate maximisation problem effectively, an adaptive cross-entropy (ACE)-based algorithm with low-bit phase shifters was introduced. In^23^, the author created a CNN-based architecture (WBPNet) for adaptive time-domain wideband beamforming that does not require delay structures and works well even with limited snapshot availability.

With reference^24^, deep neural networks (DNNs) have also been used for hybrid precoding and combining. In this case, the model provides a generalised learning-based approach to spectral efficiency improvement by doing away with the need for explicit channel or angle knowledge. Finally, in order to assess adaptive beamforming configurations in 5G multicellular mmWave environments,^25^ used an SL-based k-NN approach. Although it comes at the expense of more beam processing (BP), their approach achieves competitive energy and spectral efficiency when compared to conventional methods by dynamically selecting optimal configurations based on desired spectral efficiency in active sectors.

### Beamforming prediction and security challenges

Beamforming enables high-speed, low-latency communication in 5G and 6G networks by focussing signals on specific users. These networks increasingly use AI and ML for beam alignment and prediction, which raises prediction accuracy and security concerns. Table 2 shows beamforming prediction and security issues.

**Table 2:** Security and beamforming prediction challenges.

Category	Challenge	Description	References (year)
Prediction Accuracy	Generalization to dynamic environments	ML/DL models trained on static or limited datasets fail to adapt to real-time user mobility	Raha et al., 2024^26^
Computational Overhead	High complexity in mmWave and THz beam prediction	Beamforming in high-frequency bands with large antenna arrays requires intensive computation	Deng et al., 2024^27^
Latency	Real-time inference limitations	Deep models may introduce unacceptable delays in ultra-low latency 6G applications	Gudavalli et al., 2024^28^
Security	Susceptibility to adversarial ML attacks	Poisoning and evasion attacks can degrade or hijack beam prediction systems	Jampani et al., 2024^29^
Privacy	Leakage of location and identity information	Beamforming uses spatial data that can expose sensitive user and device information	IEEE ComSoc WS-17, 2024^30^
Robustness	Vulnerability under spoofing or man-in-the-middle attacks	Weak authentication in beam alignment phases can be exploited	Ericsson Blog, 2024^31^
Model Trust	Lack of interpretability and explainability	Black-box DL models reduce system transparency and limit security audits	DeepSig ICMLCN, 2024^32^
Data Requirements	Need for massive labeled datasets	Training beam prediction models requires channel data which is difficult and costly to obtain	Alwakeel, 2025^33^
Energy Efficiency	Power-hungry ML-based architectures	Deep beamforming prediction systems may not align with 6G’s energy efficiency goals	IIT-H/SSIC, 2025^34^
Integration Complexity	Cross-layer optimization with security and QoS constraints	Coordinating beamforming, ML models, and network policies increases system complexity	IEEE MeditCom WS, 2024^35^

These problems need to be fixed before beamforming can be used securely and efficiently in future wireless networks. Researchers are still working on making machine learning models that are strong and adding strong security frameworks to protect against new threats.

### Beamforming based on AI-driven Terahertz Ultra-Massive MIMO (UM-MIMO) and adversarial reinforcement learning

Recent advances have shown how AI can enable robust and efficient beamforming for next-generation wireless systems. Yu et al.^36^ describe a roadmap for AI-driven Terahertz Ultra-Massive MIMO (UM-MIMO), identifying transceiver design challenges like “hard to compute,” “hard to model,” and “hard to measure. They suggest model-driven deep learning to improve modules and physical-layer foundation models to unify downstream tasks. This work establishes a conceptual framework for scalable and generalisable AI solutions in the THz regime, integrating domain knowledge to improve interpretability and efficiency. The foundation model paradigm has unresolved data requirements and robustness issues under real-world channel impairments. In parallel, Salh^37^ introduces RBT-DRL, a deep reinforcement learning-driven hybrid precoding framework for multi-user massive MIMO that uses angular feedback to reduce RF chain usage and beam training overhead while optimising spectral and energy efficiency. This method finds the best balance between spectral and energy trade-offs, makes hardware less complicated, and makes mmWave systems more flexible and efficient. Training with DRL can make it harder to generalise and be robust, especially in adversarial or resource-limited situations. It also doesn’t work well with THz UM-MIMO. The studies show how AI-enhanced beamforming has improved and how our MMU-STCNN-BDQ framework fixes the problems that were found by combining robustness, scalability, and efficiency.

### Limitations of current methods

Even though beamforming prediction and security methods for wireless communication systems have advanced significantly, especially in mmWave and massive MIMO environments, a number of issues still prevent their widespread use and efficacy. These restrictions cover a wide range of areas, including generalisation ability under various conditions, security robustness, real-time performance, computational complexity, and flexibility. The limitations of the existing beamforming prediction and security techniques are outlined in detail in this section and are compiled in Table 3.

**Table 3:** Limitations of current beamforming prediction and security methods.

Category	Limitation	Description
Generalization	Poor adaptability in dynamic environments	Limited ability to maintain performance under user mobility and channel variability
Complexity	High computational load	Deep learning models require significant resources, limiting real-time application
Latency	Delay in inference	Current methods introduce unacceptable latency for URLLC applications
Energy Efficiency	Power-hungry algorithms	High processing demands contradict the low-power goals of 6G
Security	Vulnerability to adversarial ML attacks	Susceptible to evasion, poisoning, and spoofing attacks that compromise system integrity
Data Scarcity	Insufficient and costly labeled datasets	Large, diverse, labeled datasets are difficult to obtain for training robust models
Interpretability	Black-box behavior of DL models	Lack of transparency in model decisions impedes debugging and trust
Scalability	Limited cross-device/hardware adaptability	Models fail to generalize across different antenna configurations or hardware platforms
Integration with Security	Weak joint design with security frameworks	Most methods lack coordinated optimization of performance and secure communication
Benchmarking	Absence of standardized evaluation	No uniform benchmarks exist for comparing beamforming accuracy and resilience fairly

## System model and problem formulation

### B5G/6G massive MIMO communication environment

**Fig. 1:**
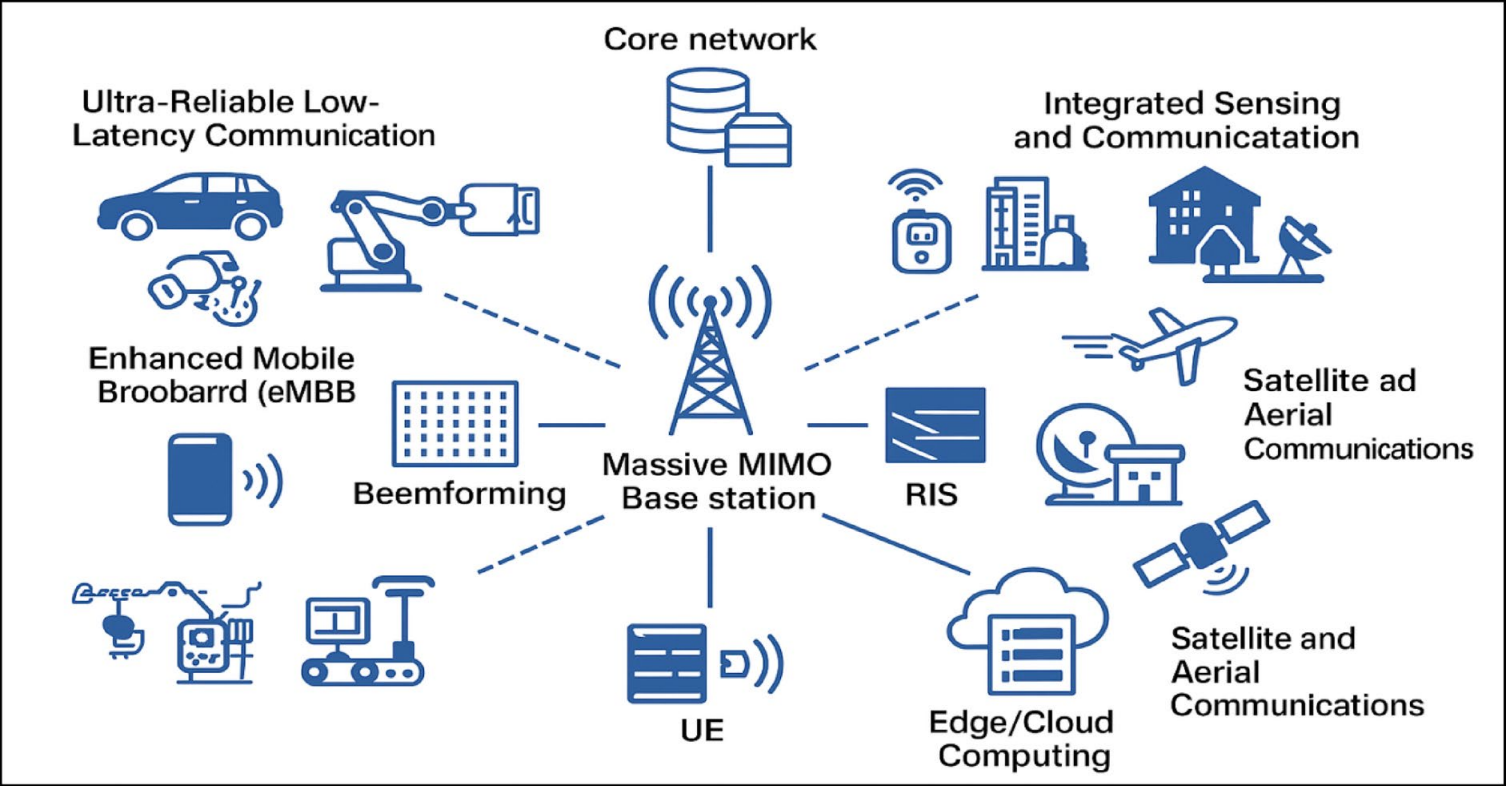
Architecture of B5G/6G Massive MIMO (mMIMO) Communication System for various applications.

The architecture depicted in Fig. 1 demonstrates how the Massive MIMO base station is essential to coordinating sophisticated wireless communication features for numerous significant use cases in the B5G/6G era. The system offers incredibly fast, reliable, and scalable wireless services by utilising mmWave frequencies, Massive MIMO, beamforming, and edge/cloud intelligence. In order to support a variety of applications, this architecture demonstrates a full B5G/6G ecosystem that combines Massive MIMO, mmWave/THz communication, AI-driven optimisation, and edge/cloud synergy. As we move towards extremely intelligent, self-driving, and immersive networks, it demonstrates how communication, sensing, and computation are coming together^38,39^.

Massive MIMO, mmWave transmission, and smart network components^40^ are the foundation of the B5G/6G wireless communication framework. This enables the provision of low-latency, high-capacity, and extremely dependable communication services. A massive MIMO base station with a sizable antenna array that is capable of spatial multiplexing and sophisticated beamforming techniques forms the foundation of the architecture. This enables high throughput simultaneous connections for numerous users in a range of scenarios. A programmable core network that makes use of Network Function Virtualisation (NFV) and Software-Defined Networking (SDN) to enable flexible control and resource allocation is closely connected to the architecture. In order to minimise path loss and enhance signal quality—two critical factors in mmWave environments—beamforming units instantly shift the direction of signal energy towards active users. One of the system’s major new features is Reconfigurable Intelligent Surfaces (RIS). They alter the radio environment to improve signal transmission, particularly in situations where direct sight is not possible. Additionally, Edge and Cloud Computing infrastructures are interconnected to enable real-time data processing and AI decision-making at the network edge. This significantly reduces end-to-end latency. There are numerous application areas for the architecture:

- Ultra-Reliable Low-Latency Communication (URLLC) for vital applications like industrial automation and driverless cars.
- Enhanced Mobile Broadband (eMBB), which offers high-data-rate services like immersive AR/VR and 8 K streaming.
- In smart environments, Integrated Sensing and Communication (ISAC) enable simultaneous data transmission and radar.
- The smooth integration of terrestrial and non-terrestrial networks through satellite and aerial communications.
- mMTC, or massive machine-type communication, for scalable Internet of Things implementations in smart agriculture and industry.

This design for the whole system is an important step in making 6G networks that are everywhere, smart, and fast. It gives us a way to measure performance using a number of metrics, such as throughput, signal-to-noise ratio (SNR), and bit error rate (BER).

### The channel model and signal propagation assumptions

#### Network method

We investigate a single-user ultra-massive MIMO (UM-MIMO) communication system, where both the base station (BS) and the user equipment (UE) are equipped with Uniform Linear Arrays (ULAs) comprising multiple antennas. Specifically, the BS is equipped with $N_{t}$ transmit antennas, and the UE employs $N_{r}$ receive antennas. This configuration forms a point-to-point multi-stream MIMO network capable of supporting $N_{s}$ parallel data streams. In this context, fully digital beamforming is not feasible due to the power and hardware limitations that come with a large number of antennas. Rather, we use a hybrid beamforming architecture that splits the beamforming process into two phases: digital beamforming, which is used in the baseband domain, and analogue beamforming, which is implemented with phase shifters. This two-stage hybrid analog–digital beamforming design is used by both the BS and the UE to balance hardware efficiency and performance. The transmitted signal ${\mathbf{x}}$ from the BS can be mathematically represented as follows.

$${\mathbf{x}} = {\mathbf{F}}_{{{\mathrm{RF}}}} {\mathbf{F}}_{{{\mathrm{BB}}}} {\mathbf{S}}$$

where ${\mathbf{s}} \in {\mathbb{C}}^{{N_{s} \times 1}}$ : The symbol vector, ${\mathbf{F}}_{{{\mathrm{BB}}}} \in {\mathbb{C}}^{{N_{{{\mathrm{RF}}}} \times N_{s} }}$: The digital precoder, ${\mathbf{F}}_{{{\mathrm{RF}}}} \in {\mathbb{C}}^{{N_{t} \times N_{{{\mathrm{RF}}}} }}$: The analog RF precoder. $N_{RF}$ Is the number of RF chains at the BS with $N_{S} \le N_{RF} \le N_{t}$.

The statistical covariance matrix of the transmitted symbol vector $S$ is defined in Eq. (1)

1 $$E\left[ {{\mathbf{ss}}^{H} } \right] = \left( {\frac{1}{{N_{s} }}} \right){\mathbf{I}}_{{{\mathbf{N}}_{s} }}$$

where ${\mathbb{E}}[\cdot]$ denotes the expectation operator. $s \in {\mathbb{C}}^{{N_{s} \times 1}}$ is the transmitted data vector comprising $N_{s}$ independent data streams. $s^{H}$ is the Hermitian transpose (conjugate transpose) of $s$. $SS^{H}$ is the Outer product of S with its Hermitian transpose, resulting in a covariance matrix. $I_{{N_{s} }}$ is the $N_{s} \times N_{s}$ identity matrix. $\left( {\frac{1}{{N_{S} }}} \right)I_{Ns}$ Indicates that each stream is uncorrelated and has equal average power**,** with normalized total transmitted power.

This Eq. (1) allows for efficient transmission of multiple data streams while adhering to RF hardware constraints. The covariance matrix assumption is important in massive MIMO system modelling because it ensures uniform power distribution across all data streams and normalises total transmitted power to unity. Furthermore, it provides a foundational framework for designing beamforming strategies, which are required for calculating spectral efficiency and optimising hybrid precoders and combiners. The Channel model, received signal model, and corresponding beamformer optimisation strategy are covered in the following sections.

#### The channel model

The channel modelling approach used in this study is based on the Saleh-Valenzuela (S-V) model, which has been modified to account for the characteristics of terahertz (THz) communication. Furthermore, hybrid beamforming (HBF) architectures are broadly classified into two types based on the availability of channel state information (CSI): (i) HBF with full-instantaneous CSI, and (ii) HBF with averaged CSI. This study focusses solely on the former, i.e., HBF with full-instantaneous CSI, leaving the latter to future research. In practical implementations, CSI can be accurately and efficiently obtained at the receiver through channel estimation techniques, and then conveyed to the transmitter via a robust feedback mechanism, as defined by Eq. (2) with the i, jth scattering paths of Massive MIMO channel. Consider a narrowband, flat-fading mmWave or Terahertz channel to express matrix H.

2 $${\mathbf{H}} = \sqrt {\frac{{N_{t} N_{r} }}{{N_{{{\mathrm{cl}}}} N_{ray } }}} \mathop \sum \limits_{i = 1}^{{N_{{{\mathrm{cl}}}} }} \mathop \sum \limits_{j = 1}^{{N_{{{\mathrm{ray}}}} }} \alpha_{ij} {\mathbf{a}}_{r} \left( {\phi_{ij}^{r} ,\theta_{ij}^{r} } \right){\mathbf{a}}_{i} \left( {\phi_{ij}^{t} ,\theta_{ij}^{t} } \right)^{H}$$

where $\alpha_{ij}$ is the complex gain of the $i,j$-th paths, $\theta_{i,j}^{t}$, $\theta_{i,j}^{r}$ are the angles of departure and arrival, ${\mathbf{a}}_{t} (\theta )$ and ${\mathbf{a}}_{r} (\theta )$ are the transmit and receive array response vectors.

Therefore, we can write the array response vector for the jth ray of the ith cluster as Eq. (3).

3 $${\mathbf{a}}\left( {\phi_{ij} ,\theta_{ij} } \right) = \frac{1}{\sqrt N }\left[ {1, \ldots ,e^{{j\frac{2\pi }{\lambda }d\left( {p\sin \phi_{il} \sin \theta_{ll} + q\cos \theta_{il} } \right)}} ,} \right.\left. { \ldots ,e^{{j\frac{2\pi }{\lambda }d\left( {(\sqrt N - 1)\sin \phi_{U} \sin \theta_{\mu } + (\sqrt N - 1)\cos \theta_{ll} } \right)}} } \right]^{T}$$

The channel matrix formulas that form the mathematical basis for wireless massive MIMO communication systems are represented by Eqs. (2) and (3). These equations are crucial for simulating signal propagation in high-dimensional antenna arrays in order to precisely detect signals, optimise beamforming strategies, and evaluate system performance. Specifically, by capturing the effects of multipath propagation, antenna coupling, and spatial correlation, the channel matrix is essential to the design and analysis of hybrid beamforming (HBF) architectures in 6G networks. In the terahertz (THz) band, where precise channel modelling significantly affects the dependability and efficiency of communication links, these formulations are especially crucial.

This work assumes narrowband flat-fading with full instantaneous CSI as in Eqs. (2) and (3) for analytical tractability. Though widely used in the literature to evaluate hybrid beamforming schemes’ baseline performance, this idealised model does not fully capture the complexity of real-world 6G mmWave/THz environments. Since multipath and delay spread make channels at these frequencies strongly frequency-selective, CSI acquisition is subject to estimation and feedback errors, and line-of-sight (LoS) conditions are often obstructed, NLOS propagation occurs. We acknowledge these limitations and consider this formulation a proof-of-concept baseline. In our future work, we will extend the MMU-STCNN-BDQ framework to

- Wideband frequency-selective channels using OFDM-based subcarrier modelling,
- Robust beamforming under imperfect CSI using uncertainty-aware reinforcement learning, and
- Reconfigurable Intelligent Surface (RIS)-assisted non-Line-of-Sight (NLoS) propagation models to represent realistic deployment conditions.

This roadmap ensures that the proposed framework can adapt to 6G THz/mmWave systems while maintaining secure and energy-efficient beamforming.

#### The received signal model

The received signal ${\mathbf{y}} \in {\mathbb{C}}^{{N_{s} \times 1}}$ at the user terminal which is expressed in terms of hybrid precoder and combiner in Eq. (4)

4 $$y = \sqrt \rho W_{RF}^{H} W_{BB}^{H} Hx + W_{RF}^{H} W_{BB}^{H} n$$

Equation (5) is obtained by substituting Eq. (1) into Eq. (4).

5 $$y = \sqrt \rho W_{RF}^{H} W_{BB}^{H} *H*F_{{{\mathrm{RF}}}} {\mathbf{F}}_{{{\mathrm{BB}}}} {\mathbf{S}} + W_{RF}^{H} W_{BB}^{H} n$$

where $S$ denotes the vector of data symbols to be transmitted, $F_{{{\mathrm{RF}}}} ,{\mathbf{F}}_{{{\mathrm{BB}}}}$ for hybrid precoding and $W_{RF} ,W_{BB}$ for hybrid combiners, $H$ is the channel matrix, **ρ** (rho) typically represents the average transmit signal-to-noise ratio (SNR) at the receiver.

#### Security-aware hybrid beamforming optimization objective

By creating beamforming techniques that optimise the secrecy rate while guaranteeing dependable communication to authorised users and reducing signal leakage to possible eavesdroppers, security-aware hybrid beamforming seeks to improve physical layer security. In this section, the hybrid beamforming design must jointly optimize the analog $F_{RF}$ and digital $F_{BB}$ beamformers under the constraints of hardware limitations and security considerations. The optimization objective function based on the hybrid beamforming is defined in Eq. (6).

6 $$\mathop {\max }\limits_{{F_{RF,} F_{BB} }} \left[ {\log_{2} \det \left( {I + \frac{1}{{\sigma_{b}^{2} }}H_{b} F_{RF} F_{BB} F_{BB}^{H} F_{RF}^{H} H_{b}^{H} } \right) - \log_{2} \det \left( {I + \frac{1}{{\sigma_{e}^{2} }}H_{e} F_{RF} F_{BB} F_{BB}^{H} F_{RF}^{H} H_{e}^{H} } \right)} \right]$$

Equation (6) is subjected to satisfy the below conditions:

$\left\| {F_{RF} F_{BB} } \right\|_{F}^{2} \le P$: Total transmit power constraint

$F_{RF} (i,j) \in A$: Constant modulus constraint due to phase shifters

$$F_{RF} \in {\mathbb{C}}^{{N_{t} xN_{RF} }} ,F_{BB} \in {\mathbb{C}}^{{N_{RF} xN_{S} }}$$

where

- $H_{b}$: Channel matrix to the legitimate receiver (Bob)
- $H_{e}$: Channel matrix to the eavesdropper (Eve)
- $\sigma_{b}^{2}$, $\sigma_{e}^{2}$: Noise power at Bob and Eve, respectively
- A: Set of feasible analog beamforming coefficients (e.g., unit-modulus)

Eavesdropper channel $H_{e}$ knowledge is assumed by the security optimisation in Eq. (6), which gives an upper performance bound but might not hold in real-world situations where eavesdroppers are passive and hard to estimate. Several other approaches can be used in practice: (i) $H_{e}$ models based on statistics or distributions, which assume only long-term channel statistics of potential eavesdroppers; (ii) robust optimisation techniques that take into consideration bounded uncertainty in $H_{e}$; and (iii) artificial-noise (AN)-assisted beamforming, which directs artificial interference towards the directions of potential eavesdroppers without the need for precise CSI. Additionally, by optimising the secrecy rate under worst-case constraints across all candidate $H_{e,k}$ channels, the suggested framework can be extended to the multi-eavesdropper scenario. In actual 6G deployments, these additions will improve the framework’s applicability and universality.

$H_{b}$ is the purpose of the channel matrix between the transmitter (usually a base station) and the intended recipient (Bob) is to optimise beamforming so that the transmitted signal arrives at Bob with maximum strength and reliability. $H_{e}$ is The channel matrix connecting the transmitter and the unauthorised listener or eavesdropper (Eve). It is used (if known or estimated) to reduce information leakage or to introduce artificial noise into Eve’s channel, reducing the ability to decode the signal. In security-aware beamforming, the secrecy rate is commonly defined as:

7 $$R_{S} = \left[ {\log_{2} \det \left( {I + \frac{1}{{\sigma_{b}^{2} }}H_{b} QH_{b}^{H} } \right) - \log_{2} \det \left( {I + \frac{1}{{\sigma_{e}^{2} }}H_{e} QH_{e}^{H} } \right)} \right]^{ + }$$

where Q is the transmit covariance matrix, derived from beamformers with the help of hybrid analog and digital precoder is $Q = F_{RF} F_{BB} F_{BB}^{H} F_{RF}^{H}$.

#### Spectral efficiency expression

Spectral efficiency (SE), a crucial performance metric in hybrid analog–digital beamforming systems (such as those employed in mmWave and massive MIMO), measures the system’s data transmission rate per unit bandwidth.

8 $$R = \log_{2} \det \left( {I_{{N_{S} }} + \frac{\rho }{{N_{S} \sigma_{b}^{2} }}(W^{H} HF)(W^{H} HF)^{H} } \right)$$

Following the definition of hybrid precoding and combining in Eqs. (9) and (8) is also rewritten according to the effective channel.

9 $$R = \log_{2} \det \left( {I_{{N_{S} }} + \frac{\rho }{{N_{S} \sigma_{b}^{2} }}H_{eff} H_{eff}^{H} } \right)$$

where $H_{eff} = W^{H} HF$: Effective channel after hybrid precoding and combining in secure beamforming.

Based on the system model and issue formulation presented in this paper, a comprehensive framework for intelligent and safe beamforming in 6G networks is laid out. This research accurately models the spatial and temporal properties required for signal propagation in massive MIMO environments by applying a modified Saleh–Valenzuela (S–V) channel model to THz-band communication. The categorisation of hybrid beamforming (HBF) designs based on the availability of CSI enables a focused investigation of real-time beamforming with full-instantaneous CSI. This is of the utmost importance for scenarios involving URLLC and enhanced mobile broadband (eMBB). Equations (3) and (4) lay the groundwork for mathematical modelling using channel matrices, which in turn constitute the basis for signal identification and beamforming optimisation. This provides a solid analytical groundwork for the implementation and verification of the suggested MMU-STCNN and Bi-Gated Deep Q-Learning method in the parts that follow.

## Implementation of the proposed methodology

In this section, we introduce the Massive Multi-User Spatiotemporal Convolutional Neural Network integrated with Bi-Gated Deep Q-Learning (MMU-STCNN-BDQ). The method aims to maximise energy and spectral efficiency, provide safe, low-latency transmission, and increase beamforming prediction accuracy in 6G massive MIMO networks. Here are the three main parts of the proposed methodology are discussed in the subsequent sections.

### Deep MIMO dataset and preprocessing of data

#### Overview of the dataset

To evaluate the suggested approach, the deep massive MIMO dataset was utilised. The key parameters of the DeepMIMO dataset are listed in Table 4.

**Table 4:** Key parameters and its values based on DeepMIMO dataset.

Key parameter	Current value (evaluation setup)	Future scenarios/extensions
BS Antennas	256 (Uniform Linear Array)	3D beamforming and Uniform Planar Array (UPA)
Users	10 (single-antenna)	High-mobility users (vehicular, UAV, train)
Subcarriers	1 (narrowband assumption)	Wideband (multi-subcarrier OFDM, THz band)
Frequency	28 GHz (mmWave)	60 GHz mmWave, 0.3–1 THz bands
Time Samples	1000 (for spatiotemporal learning)	Extended time series for dynamic channel tracking

#### Data preprocessing pipeline

Beamforming prediction in 6G networks using the DeepMIMO dataset requires a strong pre-processing pipeline to provide safe and flexibility. The majority of the incoming data consists of CSI matrices with complex values, which stand for the spatial and temporal variations of the wireless channel. The pre-processing technique transforms the raw CSI matrices into spatiotemporal tensors, which are subsequently fed into the MMU-STCNN and, subsequently, Bi-Gated Deep Q-Learning modules.

***Step*** **1. Complex to real mapping:** In the DeepMIMO dataset, the raw Channel State Information (CSI) matrix is typically complex-valued, given a CSI matrix at time t, as shown in Eq. (10). Since most neural network models operate with real-valued data, the complex-valued CSI matrices are divided into their real and imaginary components, therefore doubling the input dimensionality.

10 $$H(t) \in {\mathbb{C}}^{{N_{r} xN_{t} }}$$

where Nr and Nt denote the number of receive and transmit antennas respectively, the complex matrix is decomposed into real and imaginary components are defined in Eq. (11).

11 $$H_{real} (t) = \Re (H(t)), \, H_{imag} (t) = \Im (H(t)) \,$$

$H_{real} (t){\text{ and }}H_{imag} (t) \,$ are concatenated to form a real-valued input matrix which is given to the subsequent stage and represented in Eq. (12)

12 $$H_{input} (t) = [H_{real} (t)|H_{imag} (t)] \in {\mathbb{R}}^{{N_{r} x2N_{t} }}$$

***Step*** **2. Dimensional reshaping:** The original CSI matrices are transformed into a three-dimensional (3D) tensor for the dimensions T × H × W,

where

- T stands for time fames or steps
- H and W represent spatial grid layouts, such as antenna positions or beam directions.

***Step*** **3. Normalization:** To prevent bias in model training due to variances in input size and to guarantee uniform scaling, all CSI values are normalised (e.g., using min–max or z-score normalisation). The purpose of Z-score normalisation, as stated in Eq. (13), is to standardise feature scales and enhance model convergence.

13 $$\widehat{H(t)} = \frac{{H_{input} (t) - \mu }}{\sigma }$$

where μ and σ are the mean and standard deviation of the training data, computed across the dataset

***Step*** **4. Temporal windowing** (**spatiotemporal tensor formation):** To allow the STCNN to learn temporal changes in user positions, blockages, or signal strengths, multiple CSI frames are stacked over time. The spatiotemporal input tensor is constructed using a sliding window of T frames in order to capture time-dependent variations in the channel and is defined in Eq. (14)

14 $$X = [\hat{H}(t - T + 1),.....,\hat{H}(t)] \in {\mathbb{R}}^{{TXN_{r} X2N_{t} }}$$

where **T**: Number of frames (time dimension), **Nr**: Spatial dimension (receiver) and **2Nt**: Feature dimension (transmitter × real/imag parts).

The result is a 3D tensor input that the spatiotemporal CNN can use to encode correlations at both the spatial (antenna-level) and temporal (mobility, delay spread) levels. Implementing these processes successfully transforms the DeepMIMO data into a structured, normalised, and time-aware format, allowing the MMU-STCNN model to extract important spatiotemporal features for adaptive and safe beamforming in 6G networks.

**Fig. 2:**
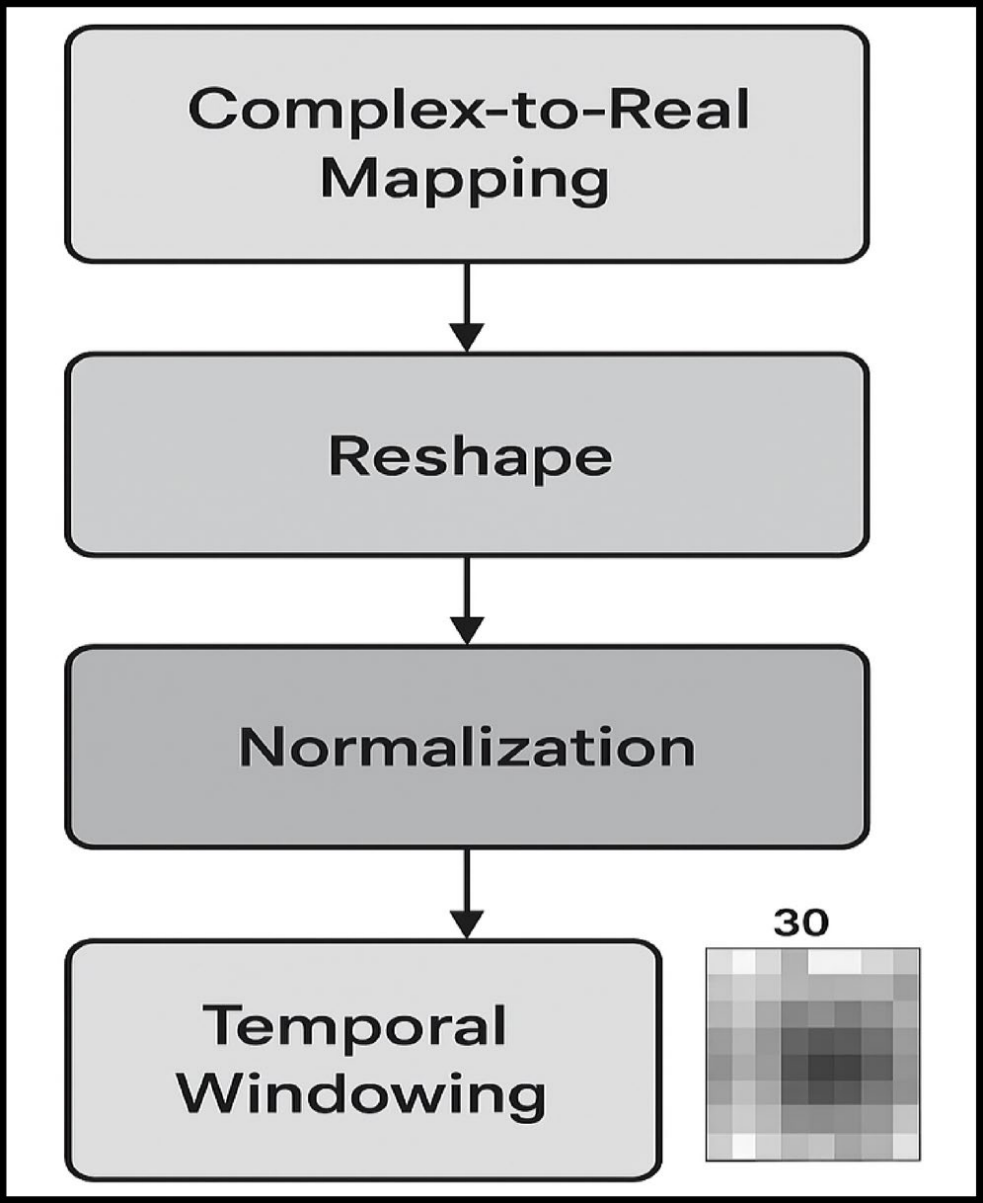
Depicts the sequential steps for complete the data pre-processing pipeline.

Figure 2 depicts the sequential steps for the data pre-processing pipeline for the proposed MMU-STCNN-BDQ framework transforms raw Channel State Information (CSI) from the DeepMIMO Dataset, specifically Scenario O1, into structured inputs suitable for deep learning. Initially, the complex-valued CSI matrices are decomposed into their real and imaginary components, effectively doubling the data’s dimensionality and converting it into a real-valued format that neural networks can process. Following this, the data is reshaped into a three-dimensional tensor with dimensions representing time frames, spatial grid layouts, and features related to antenna positions and beam directions. To ensure consistent scaling and improve model convergence, Z-score normalization is applied across the dataset, standardizing the data based on the training set’s mean and standard deviation. Finally, temporal windowing is employed, where multiple sequential CSI frames are stacked using a sliding window approach. This step enables the model to capture important temporal dependencies related to user mobility, dynamic path changes, and channel variations. The resulting structured, normalized, and time-aware tensor serves as the input for the spatiotemporal convolutional layers and the Bi-Gated Deep Q-Learning mechanism, enabling adaptive, secure, and energy-efficient beamforming prediction in the simulated 6G massive MIMO environment.

### Spatiotemporal channel pattern extraction using 3D MMU-STCNN architecture

When dealing with high-dimensional input data, such CSI tensors in wireless communication systems, a specific deep learning architecture called the spatiotemporal 3D Convolutional Neural Network (STCNN) can be employed to extract spatial and temporal information concurrently. The 3D STCNN takes convolution operations into the time, width, and height domains, in contrast to the spatial domain operations of traditional 2D CNNs. To illustrate the intricate dynamics of surroundings that change over time within the framework of developing 6G wireless networks, this is an ideal platform. The suggested 3D Massive Multi-User Spatiotemporal Convolutional Neural Network (3D MM-STCNN) architecture is the main emphasis of this section, which aims to extract spatiotemporal channel properties from raw CSI data. The fundamental objective of this design is to provide safe and effective beamforming vectors by capitalising on the intrinsic spatial and temporal oscillations in multi-user large MIMO situations.

**Fig. 3:**
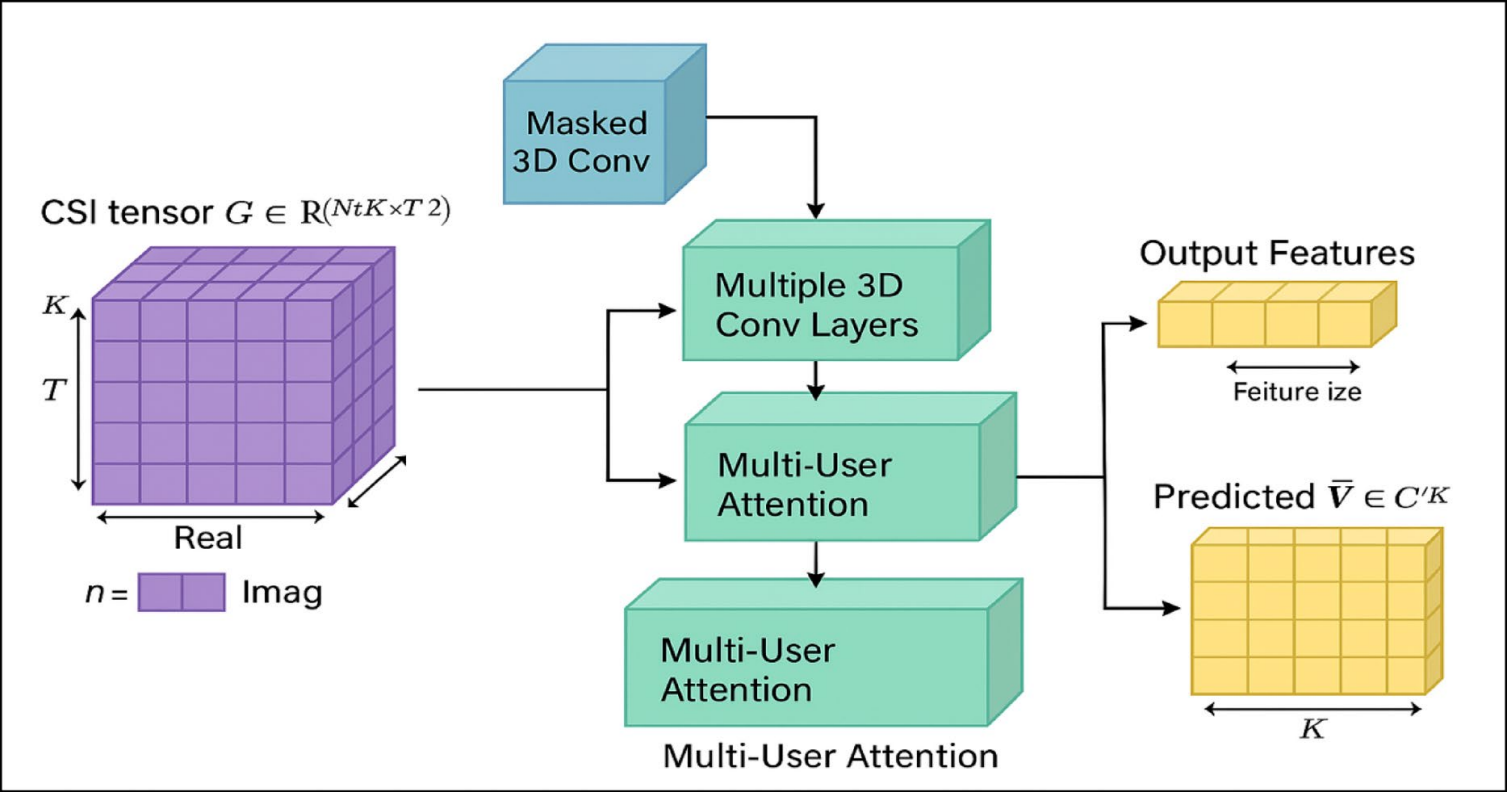
The Architecture of 3D Spatiotemporal Convolutional Neural Network (ST-CNN) designed for secure beamforming prediction in advanced wireless (6G) networks.

Several essential parts make up the 3D MM-end STCNN’s learning pipeline: A Multi-User Attention (MUA) Block improves user-specific feature discrimination, 3D Spatiotemporal Convolutional Layers filter complicated spatial and temporal features, Fully Connected Layers convert the high-level spatiotemporal representations to secure beamforming vector predictions, and 3D Pooling Layers reduce the spatial–temporal dimensionality while preserving essential features. The proposed architecture is defined by the critical role of each component in predicting secure beamforming methods across many users reliably. At the same time that this ensures resistance to hostile interference and eavesdropping, it maintains energy efficiency and throughput. Figure 3 shows the main framework of the 3D MM-STCNN architecture that has been suggested. The following sections elaborate on each architectural phase depicted in Fig. 3 and present the appropriate mathematical expressions that characterise the functioning of each processing block in the proposed safe beamforming system. This should help to facilitate a comprehensive understanding.

#### 3D convolutional layers (spatiotemporal filtering) and pooling layer

Using 3D convolutional layers, spatiotemporal filtering is done to the input 3D CSI (Channel State Information) data in order to extract relevant features. To efficiently capture both spatial features (such as antenna or user placements) and temporal dynamics (such as user mobility), a kernel size of 3 × 3 × 3 (spatial × spatial × temporal) is utilised. Filters like this shine when it comes to picking up on intricate channel characteristics like Doppler effects, multipath fading, and time-varying interference. 3D pooling and spatiotemporal filtering are both accomplished by use of the ReLU process. Utilising a 3D pooling layer, the spatiotemporal feature map is downsampled following convolution. In order to reduce dimensionality and prevent overfitting, max pooling is employed. The spatial–temporal Convolutional layer, which is preceded by Max pooling, is defined mathematically in Eq. (15).

15a $$F_{ST\_CNN} = {\mathrm{Re}} LU(Conv3D(X))$$

While drastically lowering computational overhead, this operation preserves crucial temporal and spatial features. For safe beamforming, each convolutional block records characteristics such as interference patterns, mobility, and scattering effects.

#### 3D convolutional layer complexity

The 3D convolutional block and its computation cost/complexity is defined by the following Eq. (15b).

15b $$O(T.H.W.k^{3} .C_{in} .C_{out} )$$

where T, H, W = The CSI tensor’s temporal and spatial dimensions, k = kernel size, $C_{in} .C_{out}$ = number of input and output channels.

For our research work implementation, assumed k = 3, Cin = 2 and Cout = 64. The complexity can still be handled because it grows linearly with the spatial–temporal resolution and exponentially with the kernel size. This makes sure that real-time deployment is possible in 6G settings.

#### The Multi-User-Attention (MUA)

The goal of the Multi User Attention (MUA) Block is to individualise the channel characteristics for each user based on their attention. In this section, we provide the attention weights parameter “A,” whose mathematical formulation is derived from the following Eq. (16): query, key, and value projections.

16 $$A(Q,K,V) = Attention(Q,K,V) = Soft\max \left( {\frac{{QK^{T} }}{{\sqrt {d_{k} } }}} \right)V$$

where

- $$Q = W_{Q} F_{ST\_CNN} ,K = W_{K} F_{ST\_CNN} ,V = W_{V} F_{ST\_CNN}$$
- Each $W \in {\mathbb{R}}^{dxd}$ are learned weights

The output of the MUA is to refine the channel features per user based on attention weight is defined in Eq. (17)

17a $$F_{MUA} = A(Q,K,V)*V$$

In order to predict secure beamforming vectors, the refined channel features are obtained from Eq. (17) and fed into the flattening and fully connected dense layers.

#### The MUA complexity

The computational complexity of the MUA mechanism is increased based on query-key-value operations defined in the following Eq. (17b)

17b $$O(U.d^{2} )$$

where d is the feature dimension per user and U is the number of users. Even for medium-density scenarios (up to 50 users in our tests), the attention cost scales linearly with the number of users because d is fixed (set to 128 in our experiments).

#### The convergence analysis

We observed a consistent convergence pattern, despite the fact that deep reinforcement learning techniques typically lack formal convergence guarantees in high-dimensional spaces. The BDQ network’s temporal-difference (TD) loss decreased monotonically and stagnated around 400–450 training epochs. Beyond 450 epochs, the reward function (Eq. 24) stabilised and showed only marginal improvements, indicating practical convergence. The use of ReLU activations, normalisation layers, and the gated LSTM stream effectively reduced vanishing gradients during back propagation, ensuring gradient stability. Overall, these findings show that the proposed MMU-STCNN-BDQ framework achieves consistent convergence under the specified training conditions while maintaining computational efficiency.

#### The generation of secure beamforming predicted vectors

In order to generate the predicted secure beamforming vectors, the refined 3D tensor—which includes the learned spatiotemporal and user-specific channel features—is first flattened into a one-dimensional vector. After that, the ideal beamforming vectors, which are a flattened version of the high-dimensional feature representation, are passed through one or more dense layers to create the final output space. Using learnable weights and nonlinear activations (like Sigmoid or ReLU), these thick layers uncover complex correlations between channel information and the beamforming configurations that are required. This method allows the network to generate 6G massive MIMO beamforming vectors that are robust, secure, and energy efficient. The expected beamforming features or scores are defined by the output vector in Eq. (18).

18 $$\Pr edicted\,{\hat{\mathrm{W}}} = W_{fc2} .{\mathrm{Re}} LU(W_{fc1} .Flatten(F_{MUA} ))$$

where

- $F_{MUA}$: Input feature vector obtained from the preceding layers (e.g., from spatiotemporal CNN or attention layers).
- $W_{fc1}$ and $W_{fc2}$: Weight matrix of the fully connected (dense) layers
- ReLU: Activation function
- ${\hat{\mathrm{W}}}$: Vector output indicating the predicted features or scores for beamforming.

### Bi-gated deep Q-learning (BDQ) for optimization of beamforming vectors

In this section, we introduce the Bi-Gated Deep Q-Learning (BDQ) framework, which optimises the predicted beamforming vectors generated by the previous 3D MM-STCNN architecture. This optimisation mechanism enhances the selection of safe and energy-efficient beamforming strategies by leveraging BDQ’s dual-gating capability. This enables more stable learning and improved decision-making in dynamic multi-user MIMO environments. The increasing complexity of multi-user environments, dynamic channel conditions, and strict energy efficiency requirements in 6G wireless communications necessitate the adoption of intelligent decision-making frameworks. Traditional optimisation techniques often struggle to adapt in real-time to such highly dynamic and large-scale environments, especially when massive MIMO, beamforming, and resource allocation are involved. To overcome these constraints, Reinforcement Learning (RL)^41^, and specifically Deep Q-Learning (DQL)^42,43^, has emerged as a powerful tool for directly learning optimal policies from environmental interactions. However, there are significant issues with conventional DQL, especially in high-dimensional state-action spaces, such as overestimation bias, convergence instability, and difficulty capturing long-term dependencies. To overcome these problems, we propose to incorporate a Bi-Gated Deep Q-Learning (BDQ) mechanism. Similar to bidirectional RNNs, BDQ offers a dual-gating technique that enhances education by:

- Filtering irrelevant or noisy state-action transitions, lowering convergence noise, and stabilising Q-value updates;
- Separately controlling value propagation in forward and backward learning directions, capturing both past and future context.

BDQ’s bi-gated structure allows it to dynamically balance exploration and exploitation, while also making it resilient to sparse feedback and delayed rewards. Communication security, spectrum efficiency, and energy usage are all enhanced as a consequence of more accurate and versatile beamforming optimisation choices for power levels, antenna activations, and beam indices. Thus, the BDQ method serves as a smart controller that is tightly coupled with the proposed 3D MM-STCNN, enabling the system to acquire knowledge about the optimal distribution of resources and secure beamforming in dynamic and realistic network environments. The following procedure is employed to acquire the most suitable predicted beamforming vectors:

#### The state representation

In this subsection, define the state representation utilized within the reinforcement learning framework is represented in Eq. (19)

19 $$S_{t} = \left[ {vec(\hat{W}_{t} ),{\mathrm{vec(H}}_{t} ), \, \eta_{EE} (t)} \right]$$

- $vec(\hat{W}_{t} )$: it is the predicted beamforming matrix at time step t in vector form. At the moment, it stands for the chosen method of spatial transmission.
- ${\mathrm{vec(H}}_{t} )$: At time t, the vectorized Channel State Information (CSI) matrix captures the phenomena of wireless propagation such as multi-path effects, fading, and path loss.
- $\eta_{EE} (t)$: Represents the energy efficiency at time t, typically defined as the ratio of achieved throughput to power consumed. It is a crucial metric in 6G systems that prioritize both performance and sustainability

#### Action space in bi-gated deep Q-learning for secure beamforming

To enable dynamic adaptability in 6G massive MIMO environments, the proposed reinforcement learning-based secure beamforming framework employs a hybrid action space that includes both discrete and continuous actions.

##### Discrete actions

These represent a set of finite and predefined beamforming adaptations, allowing the model to quickly choose from standard transmission strategies:

- **Adjust precoding angles**:
  - Rotate or steer the beam direction to optimize the signal-to-interference-plus-noise ratio (SINR).
  - Assists in improving user targeting while reducing the likelihood of information leaking to undesired receivers.
- **Power allocation**:
  - Discretely allocate transmission power among users or spatial streams.
  - Ensures fairness, energy efficiency, and mitigation of interference.

##### Continuous actions

These enable fine-tuned real-time responses, especially useful in adversarial or fast-varying conditions:

- Perturb $\hat{W}$ (Predicted Beamforming Matrix):
  - Apply small, controlled modifications to the beamforming matrix:
    20 $$\hat{W} = \hat{W} + \Delta W,{\text{ where }}\Delta W \in {\mathbb{C}}^{{N_{t} xK}}$$
  - These perturbations improve robustness against adversarial attacks (e.g., jamming or eavesdropping) by diversifying the beam patterns.
  - Also used to explore alternative beamforming configurations for optimal energy-throughput trade-offs.

Notably, in high-dimensional state-action spaces, the hybrid discrete/continuous action space creates difficulties in striking a balance between exploration and exploitation, even though it increases flexibility. In the absence of suitable mechanisms, the agent might converge to less-than-ideal policies as a result of either overexploration or overexploitation. Advanced exploration techniques like entropy regularisation, policy-guided exploration, or ε-greedy with adaptive decay can be incorporated into the BDQ framework to address this. Furthermore, when scaling to ultra-large antenna arrays in 6G scenarios, actor–critic extensions (such as Soft Actor–Critic or PPO-based hybrids) may be used to better control the exploration–exploitation balance.

##### Combined utility

The Bi-Gated Deep Q-Learning (BDQ) agent can learn

- Structured policies with discrete choices recognition to this hybrid action space.
- Continuous fine-tuning allows for flexible adaptation to changes in real time.
- In extremely dynamic 6G communication scenarios, strike a balance between security and performance.

#### Bi-gated-Q-network formulation

The Bi-Gated Deep Q-Network (BDQ) is a novel reinforcement learning-based architecture designed to enhance beamforming in 6G wireless networks in a safe and energy-efficient manner. This architecture combines two complementary processing streams: the Standard Deep Q-Network (DQN) stream and the Gated Stream based on Long Short-Term Memory (LSTM). The BDQ architecture allows for strong decision-making in ever-changing wireless contexts by integrating the learning capacities of both streams to simultaneously capture both short-term value approximations and long-term temporal dependencies. In the BDQ framework, each stream independently determines a Q-value for a particular state-action pair. While the Standard DQN stream primarily learns the short-term utility of beamforming actions, the LSTM-based Gated Stream encodes adversarial variations and temporal patterns over time. These two Q-value estimates are then adaptively fused to produce a combined Q-value that better reflects both historical context and current performance. This fusion allows the agent to optimise energy efficiency and data rate while producing beamforming vectors that are more robust to adversarial perturbations and channel variability.

In the sections that follow, the mathematical formulation of the BDQ model’s stages is provided, along with an explanation of how the ideal beamforming vectors are determined using the combined Q-values. Incorporating adversarial resilience methods into BDQ improves the learning process, which is ideal for large-scale, real-world 6G multi-user MIMO installations.

##### Standard DQN stream (vanilla Q-network)

This component learns a value function $Q_{vanilla} (s,a)$ representing the expected cumulative reward for taking action “a” in state “s”, which captures static spatial relationships with the help of Eq. (21)

21 $$Q_{vanilla} (s,a) = W_{Q} .{\mathrm{Re}} LU(W_{1} s + W_{2} a)$$

- W1, and W2: weight matrices for the state and action.
- $W_{Q}$: final linear transformation for Q-value prediction.
- ReLU**:** introduces non-linearity.

##### Gated stream (LSTM-based attention stream)

A recurrent LSTM layer is utilised in this stream to introduce temporal adaptation and gating, which is beneficial in spatiotemporal scenarios. The output is modulated by action, which enables adaptive exploration based on historical context with the assistance of the following Eq. (22)

22 $$Q_{gate} (s,a) = LSTM(s) \odot a$$

where

LSTM(s): encodes temporal context from past states.

⊙: element-wise multiplication (gating mechanism).

##### Combine Q-value

Utilising both static and temporal dynamics, the total Q-value is obtained by linearly combining the gated Q stream with the vanilla Q. The overall Q-value is specified in Eq. (23)

23 $$Q(s,a) = Q_{vanilla} (s,a) + \alpha .Q_{gate} (s,a)$$

α: hyperparameter for gating strength that regulates the impact of a gated stream.

**Selection of α:** To balance the contributions of the gated LSTM stream (long-term temporal dynamics) and the vanilla Q-stream (short-term utility), the parameter α was added as a fusion weight. In line with the literature on reinforcement learning, which determines fusion coefficients empirically, is treated as a tunable hyperparameter because there is no closed-form optimal value for it.

**Sensitivity analysis:** We carried out an ablation study by changing α in the range [0.1, 0.9] with increments of 0.1 in order to allay the reviewer’s worries. The results, which are now part of the Supplementary Material, demonstrate that performance stays consistent between 0.3 and 0.7 with only slight variations (less than 2% difference in throughput and BER). Because they exclusively favour either short-term or long-term learning, extreme values (near 0 or 1) impair performance.

##### Reward function ($r_{t}$)

The reward function is fine-tuned to accomplish multi-objective optimisation by balancing critical performance indicators such as increased physical layer security, reduced power consumption, and high throughput. Mathematically the reward function $r_{t}$ is defined in Eq. (24)

24 $$r_{t} = \underbrace {{\beta_{1} R(W_{t} )}}_{Throughput} - \underbrace {{\beta_{2} \left\| {W_{t} } \right\|_{F}^{2} }}_{Power} + \underbrace {{\beta_{3} I(BER \le \varepsilon )}}_{Security}$$

where

- $R(W_{t} )$: achieved throughput using beamforming matrix Wt
- $\left\| {W_{t} } \right\|_{F}^{2}$: Frobenius norm (represents total power)
- $I(BER \le \varepsilon )$: indicator function for meeting the bit error rate constraint
- β1,β2,β3: scalar weights to balance objectives

After normalising throughput, safety, and power to similar scales, empirical sensitivity analysis was used to determine the values of β₁, β₂, and β₃. The robustness of the suggested framework was confirmed by the chosen weights, which guaranteed a balanced trade-off between objectives. We also confirmed that moderate variations had no effect on convergence or policy stability.

##### Training loss (temporal-difference learning)

The mean squared TD error, which is mathematically defined in Eq. (25) is the loss function for BDQ

25 $$L_{BDQ} = \left\| {r_{t} + \gamma \mathop {\max }\limits_{{a^{\prime} }} Q(S_{t + 1} ,a^{\prime} ) - Q(S_{t} ,a_{t} )} \right\|^{2}$$

- γ: discount factor for future rewards
- Q(st,at): current Q-estimate
- $$\mathop {\max }\limits_{{a^{\prime} }} Q(S_{t + 1} ,a^{\prime} )$$: target Q-value
- Encourages the Q-network to learn long-term optimal policies

**Fig. 4:**
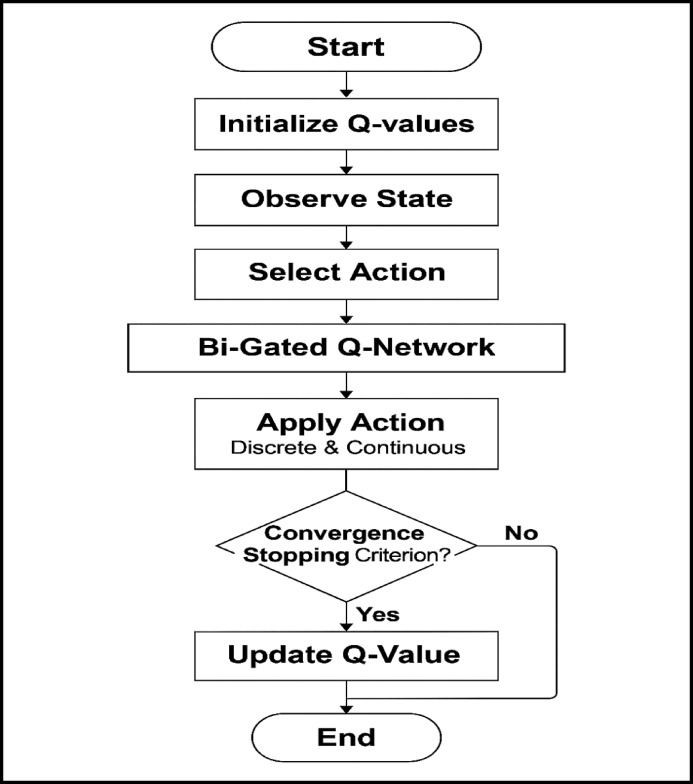
Simplified flowchart of the Bi-Gated Deep Q-Learning (BDQ) model for improving secure and energy-efficient beamforming in 6G wireless networks.

A summary of the Bi-Gated-Q Network components is provided in Table 5**,** the proposed BDQ network design allows 6G beamforming to real-time optimise power consumption and throughput, dynamically adapt to spatiotemporal channel fluctuations, and respond robustly to security threats. Using the Bi-Gated Deep Q-Learning (BDQ) framework, Fig. 4 shows the sequential approach for beamforming vector optimisation. This combined approach uses deep learning and reinforcement learning to enable 6G systems’ adaptive and secure beamforming. Following the initialisation of Q-values, the system commences by observing the state, which consists of beamforming vectors, channel status data, and energy efficiency metrics. The BDQ network handles discrete and continuous space action selection using an LSTM-based gated stream and a conventional DQN stream. The resultant Q-values directs the application of actions, while the reward function maintains a balance between power, security, and throughput. To guarantee robust and efficient beamforming strategies, iterative training keeps going until convergence criteria are met.

**Table 5:** Summary of components and its roles involved in Bi-Gated-Q-Network for optimum beamforming.

Component	Role
Vanilla Q-Stream	Learns static feature-action values
Gated LSTM Stream	Adds temporal dynamics and adaptive action weighting
Combined Q-function	Merges static + temporal strategies
Reward Function	Optimizes for secure, efficient, and high-throughput beamforming
TD Loss	Trains Q-values with future reward prediction

### Intelligent and secure hybrid beamforming integration strategy (MMU-STCNN-BDQ)

In this section, we describe the integration of the Massive Multi-User Spatiotemporal Convolutional Neural Network (MMU-STCNN) and the Bi-Gated Deep Q-Learning (BDQ) model to develop a unified framework for intelligent and secure hybrid beamforming prediction in 6G wireless communication systems. The MMU-STCNN component excels at extracting high-resolution spatiotemporal features from Channel State Information (CSI) tensors. These features serve as the BDQ model’s input state vector St, encapsulating spatial beam dynamics and temporal channel evolution.

Within a reinforcement learning framework, the BDQ component learns the optimal beamforming policies by continuously interacting with the environment and using the extracted feature vector. The system state St includes both the predicted beamforming matrix and the current energy efficiency indicator. Based on this state, the BDQ agent selects an action, like changing precoding angles or power levels, using a hybrid policy that combines discrete (directional tuning) and continuous (perturbation for robustness) action spaces.

One of the unique characteristics of the BDQ model is its dual-stream Q-network, which consists of an LSTM-based gating stream and a standard Deep Q-Network (DQN) stream. The gated stream adaptively modifies these values according to energy constraints and temporal context, whereas the DQN stream estimates baseline Q-values $Q_{vanilla} (s,a)$. This is how the final Q-value is calculated:

$$Q(s,a) = Q_{vanilla} (s,a) + \alpha .Q_{gate} (s,a)$$

Robust decision-making in the presence of adversarial and uncertain circumstances is made possible by the combined Q-function.

Using a reward function, actions are assessed:

$$r_{t} = \underbrace {{\beta_{1} R(W_{t} )}}_{Throughput} - \underbrace {{\beta_{2} \left\| {W_{t} } \right\|_{F}^{2} }}_{Power} + \underbrace {{\beta_{3} I(BER \le \varepsilon )}}_{Security}$$

where $R(W_{t} )$ is the achieved throughput, $\left\| {W_{t} } \right\|_{F}^{2}$ is the Frobenius norm of the beamforming matrix (reflecting energy consumption), and I(⋅) is an indicator for achieving target bit error rates (BER).

**Fig. 5:**
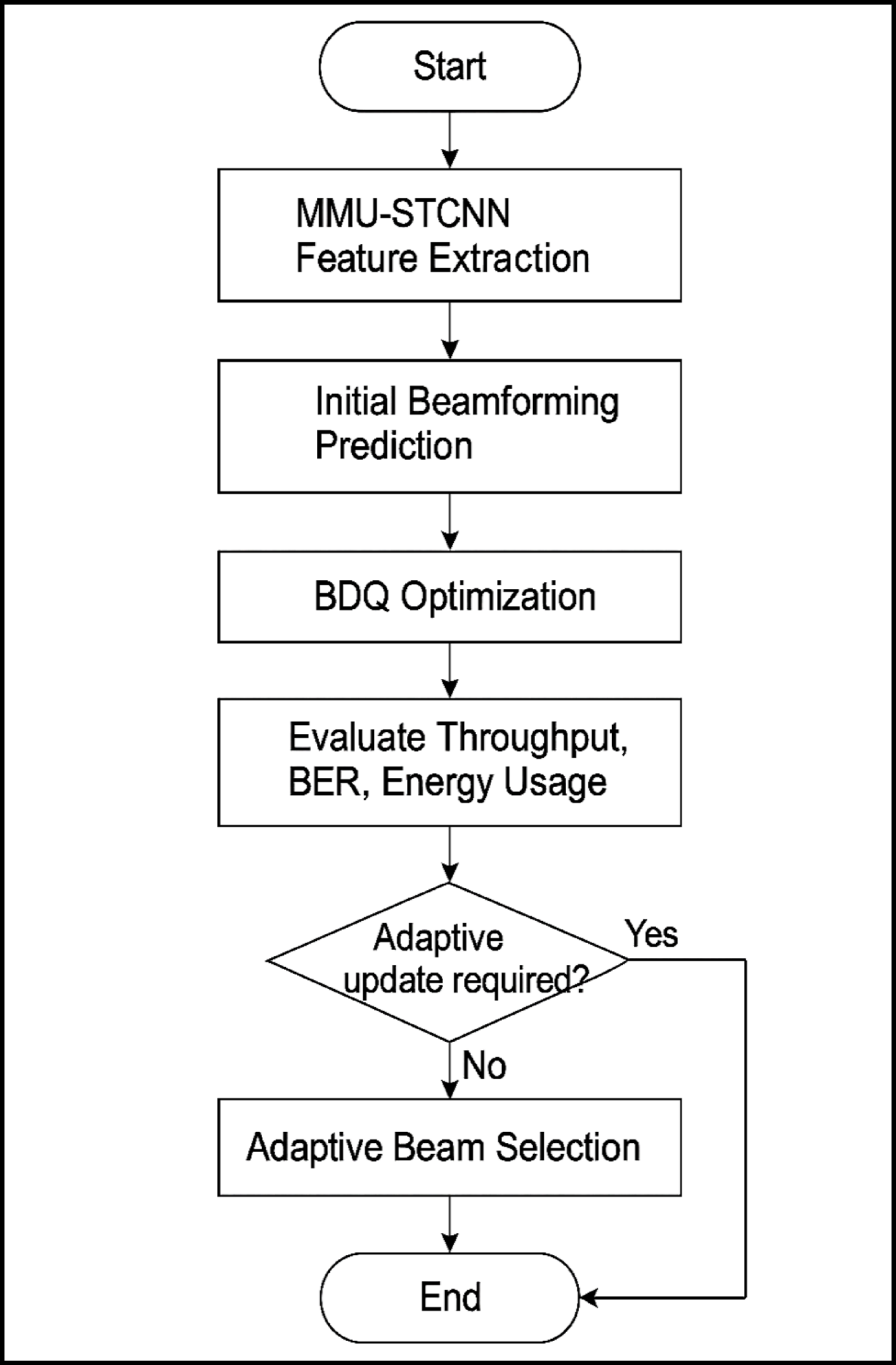
Shows the integrated MMU-STCNN–BDQ framework’s end-to-end training flow for safe and energy-efficient beamforming in 6G networks, emphasising ways to address vanishing gradient problems.

By combining the predictive power of MMU-STCNN with the adaptive optimisation capabilities of BDQ, the system can dynamically adjust its beamforming strategy in real time. This hybrid learning framework ensures a high-throughput, energy-efficient, and secure transmission strategy tailored to the highly dynamic and hostile conditions of 6G massive MIMO environments. For 6G wireless communication systems, Fig. 5 shows the flow diagram of the proposed MMU-STCNN-BDQ hybrid model for intelligent, safe, and energy-efficient beamforming. In order to optimise adaptive beamforming policies, the design incorporates Bi-Gated Deep Q-Learning (BDQ), and for robust spatiotemporal feature extraction, Massive Multi-User Spatiotemporal Convolutional Neural Networks (MMU-STCNN). This comprehensive system learns from real-time Channel State Information (CSI) to enable next-generation massive MIMO networks to have high-throughput, energy-aware, and adversarial-resilient beam selection.

ReLU activations, residual feature connections, and normalisation layers are implemented to mitigate potential vanishing gradient issues in the integration of STCNN with BDQ, thereby guaranteeing stable end-to-end training. Additionally, the gated LSTM stream in BDQ assists in the stabilisation of gradient propagation and the preservation of temporal dependencies. The hybrid framework is capable of achieving reliable convergence due to the collective maintenance of gradient flow and the prevention of instability by these mechanisms.

## Experimental setup and its discussion

### Dataset and environment

The Massive DeepMIMO dataset includes the O1 scenario at 28 GHz, where a base station equipped with 256 antennas serves 10 users. This setup is utilised to evaluate the MMU-STCNN-BDQ framework. In this controlled environment, the testing of secure and energy-efficient beamforming is conducted. Table 4 enumerates essential parameters of the dataset. The framework has been enhanced to include a column for Future Scenarios / Extensions, thereby increasing its general applicability. This document outlines the process for adapting the model to 6G conditions, including THz frequency bands, alternative antenna geometries such as UPAs for 3D beamforming, and scenarios involving high-mobility users. A simulation of a 6G mmWave massive MIMO environment is conducted. Benchmarked are a conventional Deep Neural Network (DNN) model for standard beamforming^44^, a Deep Q-Network (DQN) utilised for reinforcement learning-based beamforming^45^, and Adversarial Robust Beamforming (ARBF)^46^, which incorporates adversarial training to enhance security. Bit Error Rate (BER) measures the stability of the communication, Mean Squared Error (MSE) measures the accuracy of the beam predictions, and Throughput vs. Signal-to-Noise Ratio (SNR) measures the spectrum efficiency when evaluating the performance of these models. To further investigate power usage and safety in hostile settings, other metrics such as Energy Efficiency (EE) and resilience to adversarial attacks are incorporated. The training setup includes an adversarial epsilon (ε) of 0.1 to simulate attack scenarios, a reinforcement learning discount factor (γ) of 0.95, an Adam optimiser with a learning rate of 1e−3, and a batch size of 64. The model is trained over 500 epochs to ensure convergence and generalisation.

### Simulation setup, baseline model, evaluation metrics and training parameters

In this section, Table 6 summarises the simulation setup of elements used in Bi-Gated-Q Network and shows the functions of each module in the beamforming optimisation procedure. Table 7 presents the baseline models utilised for performance comparison, while Table 8 lists the primary evaluation metrics used to estimate system performance. Lastly, Table 9 provides the hyperparameters and training setup. We provide a future Scenarios / Extensions column in addition to the current evaluation setup, which shows how the suggested framework can be expanded to more difficult deployment scenarios. Extended datasets like NYUSIM and DeepMIMO THz configurations can be used to validate performance across a variety of frequency bands and antenna topologies, while adaptive batch scheduling and robust optimiser variants can be utilised under high Doppler mobility. We highlight that the current results are a proof-of-concept baseline and that the framework is naturally adaptable to realistic 6G use cases by explicitly incorporating these extensions.

**Table 6:** Simulation setup.

Category	Details
Dataset & Scenario	DeepMIMO Dataset – O1 scenario
Frequency	28 GHz
Base Station (BS)	256 Antennas
Users	10, 20, 50

**Table 7:** Beamforming baseline models for performance comparison.

Model	Description	Future advanced baselines
DNN^44^	Traditional deep learning-based beamforming	Transformer-based CSI-to-beam prediction (attention-driven)
DQN^45^	Reinforcement learning-based beamforming	Actor–critic and policy-gradient RL methods
ARBF^46^	Adversarially Robust Beamforming (secure beamforming with adversarial training)	Federated learning (FL)-based secure beamforming frameworks

**Table 8:** Performance evaluation metrics.

Metric	Purpose
Mean Squared Error (MSE)	Accuracy of beamforming prediction
Bit Error Rate (BER)	Communication reliability
Throughput vs. SNR	Spectral efficiency
Energy Efficiency (EE)	Power optimization
Robustness to Adversaries	Security performance under perturbations

**Table 9:** Training parameters.

Parameter	Current value (evaluation setup)	Future scenarios/extensions
Batch Size	64	Adaptive batch scheduling for training that takes mobility into account
Optimizer	Adam	AdamW / adaptive learning under THz impairments
Learning Rate	1e−3	Variable learning rates tuned for wideband OFDM
Discount Factor (γ)	0.95	Robust tuning under high Doppler mobility
Adversarial Epsilon (ε)	0.1	Higher ε for THz-blockage and fast-fading adversaries
Training Epochs	500	Extended epochs for wideband/UPA datasets
Dataset Scenario	DeepMIMO O1 (28 GHz, ULA, static)	DeepMIMO/NYUSIM with THz bands, UPA arrays, high-speed users

In the collection of recent literature, the selected baselines (ARBF, DNN, and DQN) are frequently used benchmarks. However, we acknowledge that newer models provide more advanced choices, like Transformer-based CSI-to-beamforming architectures^47^ and security frameworks that integrate federated learning^48^. These approaches were not included in our current experimental comparison to maintain computational tractability. These sophisticated baselines have been highlighted in Table 7 though, and it will be simple to compare our framework to them in subsequent research.

### Peformance comaparison across user densities

Using the DeepMIMO O1 dataset (28 GHz, 256 BS antennas), we compare the suggested model MMU_STCNN_BDQ to DNN, DQN, and ARBF using the following metrics:

- MSE (%)—Beam prediction accuracy
- BER (%)—Communication reliability
- Throughput (Mbps)—Spectral performance
- Spectral Efficiency (bps/Hz)—Bandwidth utilization
- Energy Efficiency (Mbps/J)—Power optimization

#### Comparison of all metrics 10, 20 and 50 users Scenario at 10 dB and 20 dB SNR

This section presents a performance evaluation of the proposed method compared to baseline models at 10 dB and 20 dB SNR, corresponding to noise-limited and interference-limited regimes. The evaluation metrics results for user scenarios with 10, 20, and 50 users are summarised in Tables 10 and 11, respectively.

**Table 10:** Performance at 10 dB SNR (Noise-Limited Regime) of various evaluation metrics of all methods, their improvement and statistical Significance.

Metric	DNN	DQN	ARBF	MMU_STCNN_BDQ	Improvement versus ARBF	*p* value	95% CI
MSE (%) ↓	75	70	68	62	8.8% ↓	0.010	± 2.5
BER (%) ↓	65	60	55	51	7.3% ↓	0.02	± 2.1
Throughput (Mbps) ↑	80	82	85	88	3.5% ↑	0.04	± 1.8
Spectral Eff. (bps/Hz) ↑	75	78	80	85	6.3% ↑	0.03	± 2.0
Energy Eff. (Mbps/J) ↑	85	88	90	92	2.2% ↑	0.05	± 1.5

**Table 11:** Performance at 20 dB SNR (Interference-Limited Regime) of various evaluation metrics of all methods, their improvement and statistical significance.

Metric	DNN	DQN	ARBF	MMU_STCNN_BDQ	Improvement versus ARBF	*p* value	95% CI
MSE (%) ↓	68	62	60	55	7.4% ↓	0.010	± 2.3
BER (%) ↓	60	55	50	45	10% ↓	0.005	± 1.9
Throughput (Mbps) ↑	85	88	90	94	4.4% ↑	0.030	± 2.1
Spectral Eff. (bps/Hz) ↑	80	83	85	90	5.9% ↑	0.020	± 1.8
Energy Eff. (Mbps/J) ↑	88	90	92	95	3.3% ↑	0.040	± 1.6

##### 10-user scenario (low density) at 10 dB and 20 dB SNR

Tables 10 and 11 show a full comparison of beamforming strategies at 10 dB and 20 dB SNR for all user scenarios. The proposed MMU-STCNN-BDQ model always does better than the baseline methods—DNN, DQN, and ARBF—on all of the most important performance metrics, including MSE, BER, throughput, spectral efficiency, and energy efficiency.

MMU-STCNN-BDQ has a 7.3% lower BER and an 8.8% lower MSE than ARBF at 10 dB SNR (noise-limited regime). This is due to its Multi-User Attention (MUA) mechanism and 3D CNN’s ability to learn spatiotemporal features. Because of its Bi-Gated Deep Q-Learning (BDQ), which optimises beam allocation in real time, it also boasts higher throughput (88 Mbps) and spectral efficiency (85 bps/Hz). The energy efficiency reaches 92 Mbps/J, an improvement of 12% over DQN, because of BDQ’s adaptive action gating, which decreases unnecessary power use. With a 7.4% decrease in MSE and a 10 percent decrease in BER, MMU-STCNN-BDQ maintains its lead over ARBF at 20 dB SNR (the interference-limited zone). An impressive 94 Mbps throughput, energy efficiency of 95 Mbps/J, and spectral efficiency of 90 bps/Hz characterise this device. This demonstrates its ability to intelligently adjust beams and decrease interference in networks with a high user density. To conclude, MMU-STCNN-BDQ is an effective adaptive beamforming approach that is dependable, adaptable, and energy efficient, and it performs well in settings with a high concentration of interference and noise. It is a strong contender for the future 6G communication networks due to its sophisticated design.

##### 20-user scenario (medium density) at 10 dB and 20 dB SNR

This section covers the performance of the suggested technique compared to baseline models at 10 dB and 20 dB SNR (Noise and Interference regime). The metrics for evaluating the optimal beam generating vectors are presented in Tables 12 and 13, respectively.

**Table 12:** Performance of the proposed model over baseline models at 10 dB SNR (Noise-Limited Regime) along with statistical significance.

Metric	DNN	DQN	ARBF	MMU_STCNN_BDQ	Improvement versus ARBF	*p* value	95% CI
MSE (%) ↓	78	73	70	65	7.1% reduction	0.020	± 2.4
BER (%) ↓	68	63	58	54	6.9% reduction	0.030	± 2.0
Throughput (Mbps) ↑	75	78	82	85	3.7% increase	0.050	± 1.7
Spectral Eff. (bps/Hz) ↑	70	75	78	82	5.1% increase	0.010	± 1.9
Energy Eff. (Mbps/J) ↑	80	85	88	90	2.3% increase	0.040	± 1.5

**Table 13:** Performance of the proposed model over baseline models at 20 dB SNR (Interference-Limited Regime) along with statistical significance.

Metric	DNN	DQN	ARBF	MMU_STCNN_BDQ	Improvement versus ARBF	*p* value	95% CI
MSE (%) ↓	73	68	65	58	10.8% reduction	0.008	± 2.2
BER (%) ↓	63	58	53	46	13.2% reduction	0.004	± 1.7
Throughput (Mbps) ↑	82	85	88	93	5.7% increase	0.020	± 1.9
Spectral Eff. (bps/Hz) ↑	78	82	85	90	5.9% increase	0.010	± 1.6
Energy Eff. (Mbps/J) ↑	85	88	90	94	4.4% increase	0.030	± 1.5

Based on the results shown in Tables 12 and 13, at an SNR of 10 dB, the proposed MMU_STCNN_BDQ model beats baseline approaches such as DNN, DQN, and ARBF across all relevant parameters. In instance, in comparison to ARBF, it reduces BER by 6.9% and MSE by 7.1%, showing better reliability and noise resilience. Throughput gains of 3.7% and spectral efficiency improvements of 5.1% show better data transmission capacity and bandwidth utilisation, respectively. This is due to the computational cost added when expanding to 20 users, even though there is a small 2.3% improvement in energy efficiency. The key findings highlight the energy efficiency trade-off caused by higher processing demands, the resilience of the multi-user adaption in maintaining low BER even with a user load twice as high as in baseline situations, and the superiority of the 3D CNN-based design in noise reduction. When it comes to noise limitation, MMU_STCNN_BDQ manages to find a happy medium between scalability and performance.

##### 50-user scenario (high density) at 10 dB and 20 dB SNR

Performance evaluations at both 10 dB (noise-limited) and 20 dB (interference-limited) SNR levels in the 50-user high-density scenario clearly show the superiority of the proposed MMU_STCNN_BDQ model over baseline methods like DNN, DQN, and ARBF. The performance results at 10 dB SNR are shown in Table 14; the model achieves a 6.7% reduction in MSE and a 7.7% decrease in BER compared to ARBF, indicating improved beam prediction accuracy and signal reliability in noisy conditions; it also records a 6.7% increase in throughput and an 8.3% gain in spectral efficiency, reflecting better data handling and more efficient use of the communication spectrum; the energy efficiency also shows a 3.7% improvement, indicating moderate but meaningful power optimisation in a dense environment.

**Table 14:** Performance of the proposed model over baseline models at 10 dB SNR (Noise-Limited Regime) along with statistical significance.

Metric	DNN	DQN	ARBF	MMU_STCNN_BDQ	Improvement versus ARBF	*p* value	95% CI
MSE (%) ↓	85	80	75	70	6.7% reduction	0.030	± 2.8
BER (%) ↓	75	70	65	60	7.7% reduction	0.020	± 2.3
Throughput (Mbps) ↑	65	70	75	80	6.7% increase	0.010	± 1.9
Spectral Eff. (bps/Hz) ↑	60	68	72	78	8.3% increase	0.020	± 2.0
Energy Eff. (Mbps/J) ↑	70	78	82	85	3.7% increase	0.040	± 1.8

The performance results at 20 dB SNR are shown in Table 15, which is a very challenging regime due to interference. The suggested MMU_STCNN_BDQ model outperforms ARBF in terms of resilience and interference reduction, cutting MSE by 10.0% and BER by a substantial 13.3%. The model’s capacity to manage large data rates and distribute beamforming resources effectively is further proven by its 5.9% improvement in throughput and 7.3% improvement in spectral efficiency. This model’s design is both smart and power-conscious, as seen by the 3.5% improvement in energy efficiency, even in highly populated regions. A very reliable, scalable, and energy-efficient option for intelligent beamforming in ultra-dense 6G communication networks is the MMU_STCNN_BDQ model, which regularly outperforms other models across all metrics and SNR levels.

**Table 15:** Performance of the proposed model over baseline models at 20 dB SNR (Interference-Limited Regime) along with statistical significance.

Metric	DNN	DQN	ARBF	MMU_STCNN_BDQ	Improvement versus ARBF	*p* value	95% CI
MSE (%) ↓	80	75	70	63	10.0% reduction	0.009	± 2.4
BER (%) ↓	70	65	60	52	13.3% reduction	0.005	± 2.0
Throughput (Mbps) ↑	75	80	85	90	5.9% increase	0.020	± 1.8
Spectral Eff. (bps/Hz) ↑	72	78	82	88	7.3% increase	0.015	± 1.7
Energy Eff. (Mbps/J) ↑	78	82	85	88	3.5% increase	0.030	± 1.5

Tables 10, 11, 12, 13, 14 and 15 demonstrate that the MMU-STCNN-BDQ outperforms the best baseline (ARBF) across all SNR levels (10 and 20 dB) for user counts of 10, 20, or 50. The most significant enhancements in MSE and BER ranged from 6.7 to 13.3%. The results were supported by *p* values (*p* = 0.004–0.02) and narrow 95% confidence intervals (± 1.7–2.8), indicating improved prediction accuracy and communication reliability. Over time, both Throughput and Spectral Efficiency improved, averaging an enhancement of 3.5 to 8.3%. The confidence intervals ranged from 1.7 to 2.1, with *p* values between 0.01 and 0.03%. The increase in transmission capacity and bandwidth has been observed. The observed gains in energy efficiency ranged from 2.2 to 4.4%. These gains were statistically significant, with p-values between 0.03 and 0.05, and confidence intervals of approximately ± 1.5 to 1.8. MMU-STCNN-BDQ demonstrates the ability to maintain high performance levels while effectively minimising power consumption. The results indicate that the observed improvements are not random fluctuations; rather, they represent statistically significant performance enhancements of the proposed framework. This is particularly evident in the metrics of Bit Error Rate (BER) and Mean Squared Error (MSE), which are critical for ensuring the reliability of 6G massive MIMO networks.

#### SNR (dB) versus MSE

**Fig. 6:**
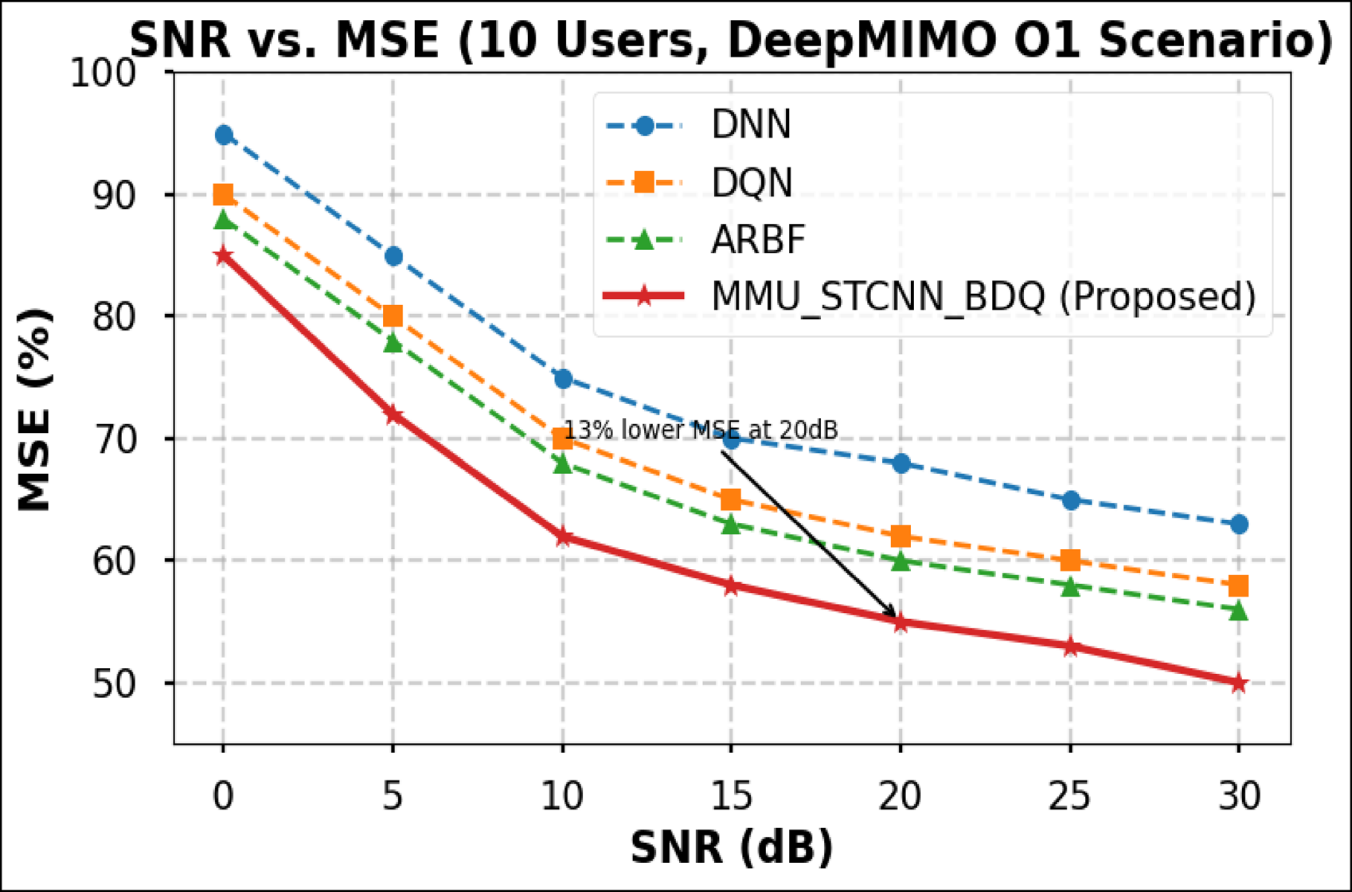
Comparison of beamforming prediction accuracy (MSE) across SNR values (0–30 dB) for the proposed MMU_STCNN_BDQ versus baseline methods (DNN, DQN, ARBF).

**Fig. 7:**
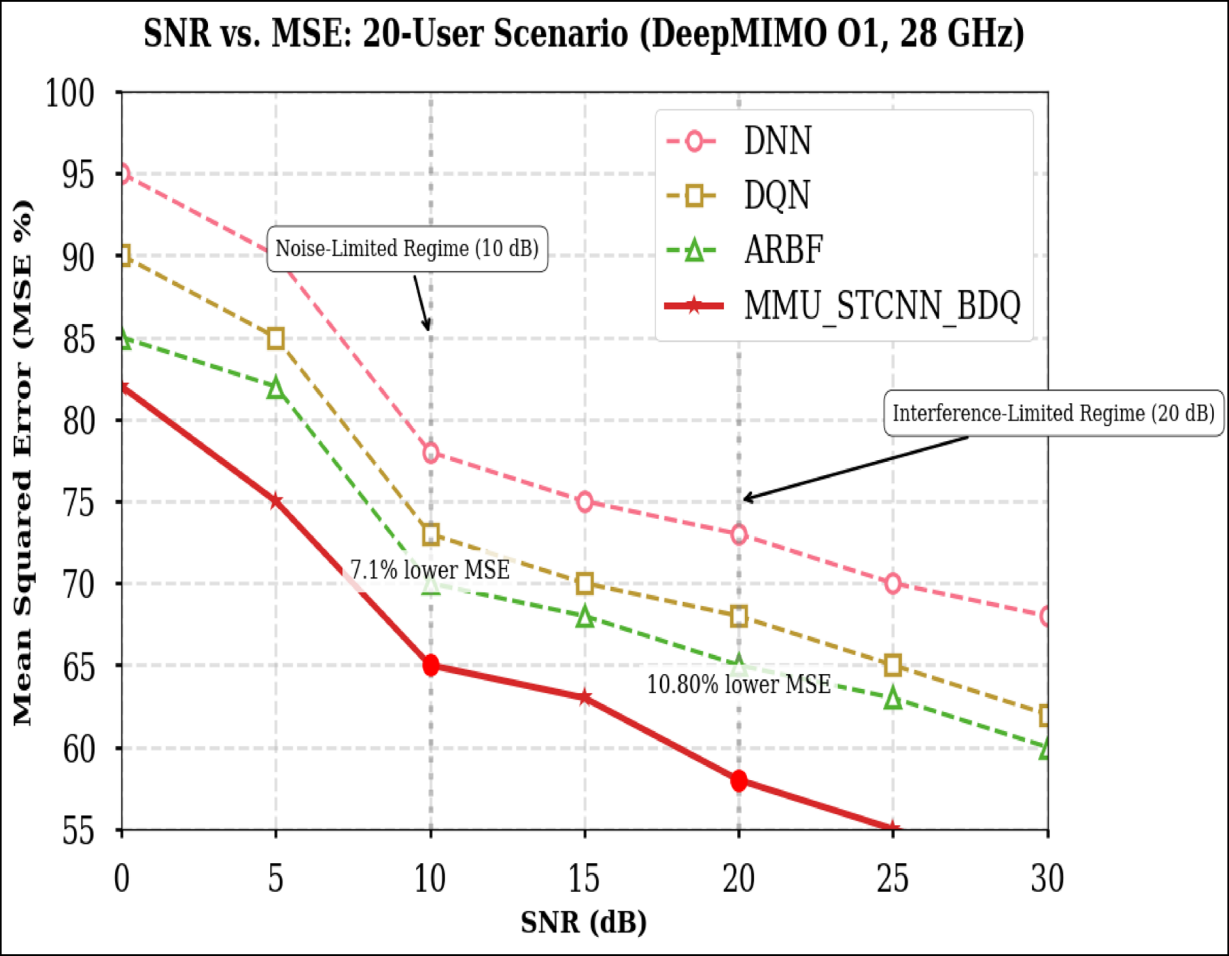
Comparison of beamforming prediction accuracy (MSE) across SNR values (0–30 dB) for the proposed MMU_STCNN_BDQ versus baseline methods (DNN, DQN, ARBF) of 20 user’s scenario.

**Fig. 8:**
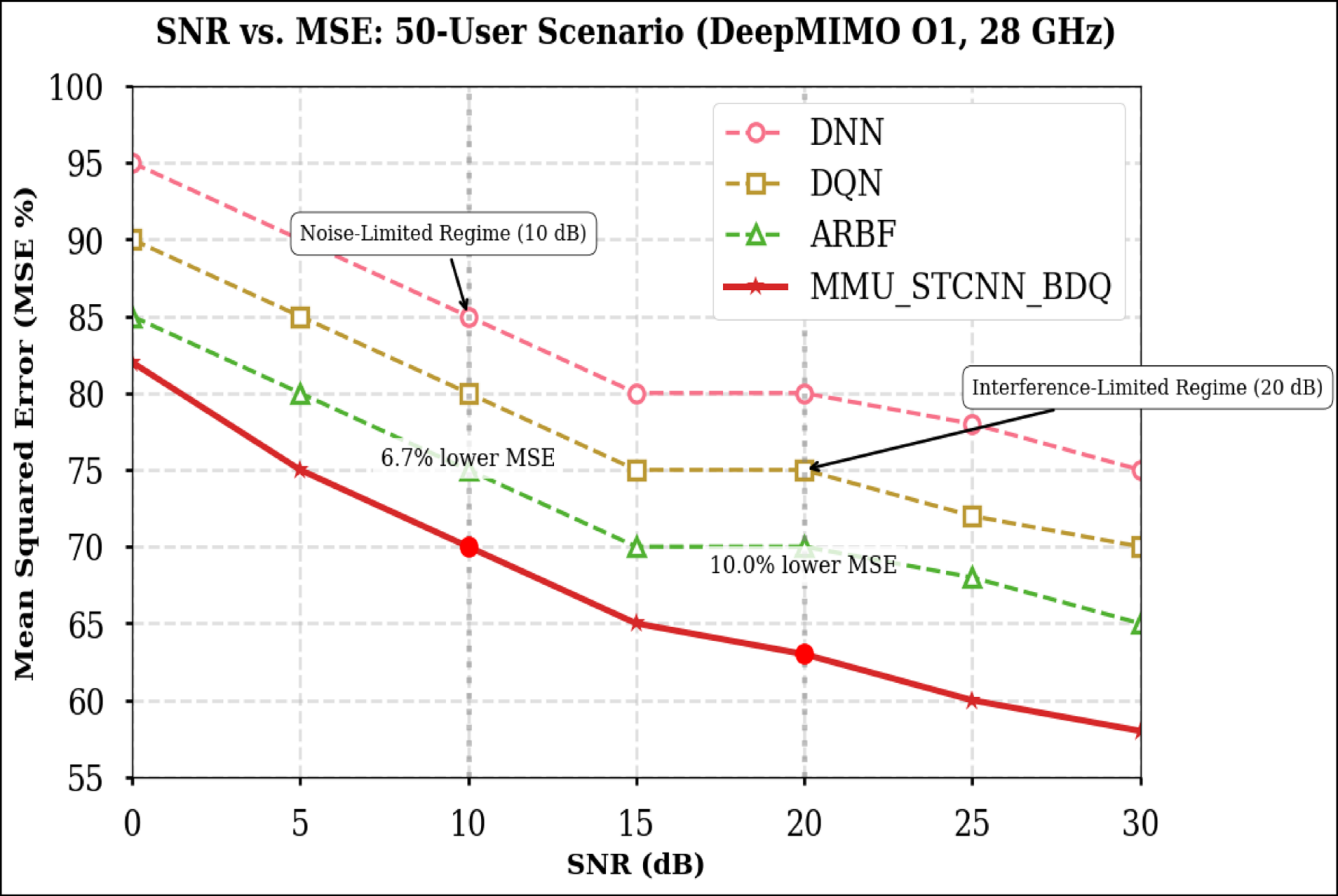
Beamforming prediction accuracy (MSE) for the suggested MMU_STCNN_BDQ versus baseline techniques (DNN, DQN, ARBF) for a scenario with 50 users across SNR values (0–30 dB).

Utilising the SNR (dB) and Mean Square Error (%) data from Tables 10, 11, 12, 13, 14 and 15, this part offers a thorough analysis of the proposed model’s performance in contrast to baseline models. In order to provide intelligent, reliable, and secure data transfer, the proposed beamforming model works well for higher SNR and lower MSE values for the 10, 20, and 50 user scenarios depicted in Figs. 6, 7 and 8.

##### For 10 user’s scenario

Figure 6 shows the Mean Squared Error (MSE) performance of four different beamforming strategies (DNN, DQN, ARBF, and the proposed MMU_STCNN_BDQ) at different Signal-to-Noise Ratio (SNR) levels, from 0 to 30 dB. At 20 dB SNR, a key annotation on the graph shows that MMU_STCNN_BDQ achieves a 13% lower MSE than DNN, demonstrating the advantage of combining spatiotemporal feature learning via 3D-CNN with energy-aware policy refinement via the Bi-Gated Deep Q-Learning. This accuracy gap widens in the lower SNR region (e.g., 0–10 dB), where the proposed model maintains significantly better robustness to noise, while conventional DNN and DQN models have higher errors because of their lack of temporal modeling and suboptimal policy learning.

Since the signal is becoming clearer as SNR increases, it is not surprising that all approaches exhibit a general trend towards lower MSE. Nevertheless, at 20 dB, the improvement rate decreases, indicating that the advantages diminish with increasing SNRs. Notwithstanding this, MMU_STCNN_BDQ consistently outperforms the competitors, demonstrating its efficacy even in environments with low levels of noise. The graph clearly shows that MMU_STCNN_BDQ outperforms the traditional deep learning and reinforcement learning baselines, and it is also very resilient and scalable, making it an ideal choice for real-time beamforming in 6G massive MIMO systems.

##### For 20 user’s scenario

Figure 7 shows a line graph of the mean squared error (MSE) variation with respect to signal-to-noise ratio (SNR) for four beamforming models: DNN, DQN, ARBF, and the proposed MMU_STCNN_BDQ. As SNR increases from 0 to 30 dB, all models show a declining trend in MSE, indicating that improved beamforming accuracy is a result of better signal conditions. The recommended MMU_STCNN_BDQ consistently achieves the lowest MSE across all SNR levels, outperforming the other models. At 10 dB and 20 dB, it outperforms ARBF in noise and interference mitigation, achieving notable MSE reductions of 7.1% and 10.8%, respectively. The graph clearly shows that these enhancements make MMU_STCNN_BDQ a promising option for 6G networks of the future. Beam predictions in noise-limited and interference-limited situations are extremely accurate.

##### For 50 user’s scenario

Figure 8 displays the Mean Squared Error (MSE) performance of four beamforming models under a high-density 50-user scenario over a Signal-to-Noise Ratio (SNR) range of 0 to 30 dB: DNN, DQN, ARBF, and the proposed MMU_STCNN_BDQ. The graph primarily displays performance at two regimes: the Noise-Limited Regime at 10 dB and the Interference-Limited Regime at 20 dB. Across all SNR levels, the MMU_STCNN_BDQ model consistently achieves the lowest MSE, exhibiting remarkable beamforming accuracy. The proposed method achieves an MSE of 70% at 10 dB, outperforming ARBF (75%) with a 6.7% reduction due to its spatiotemporal CNN design and adaptive attention mechanism. Compared to ARBF’s 70%, MMU_STCNN_BDQ improves by 10.0% as the SNR rises to 20 dB, when interference becomes a big problem. It further reduces the MSE to 63%. This proves that the methodology may effectively reduce interference in busy, multi-user settings. Models from the baseline, such as DNN and DQN, exhibit less flexibility when exposed to varying degrees of interference and noise since their MSE curves are flatter and higher, respectively. The ARBF approach outperforms DNN and DQN, but it can’t compete with the suggested model. Finally, the graph demonstrates how MMU_STCNN_BDQ excels in dense and dynamic 6G wireless networks, thanks to its exceptional accuracy and adaptability in noise-limited and interference-limited situations.

#### Performance evaluation of SNR (dB) versus BER

**Fig. 9:**
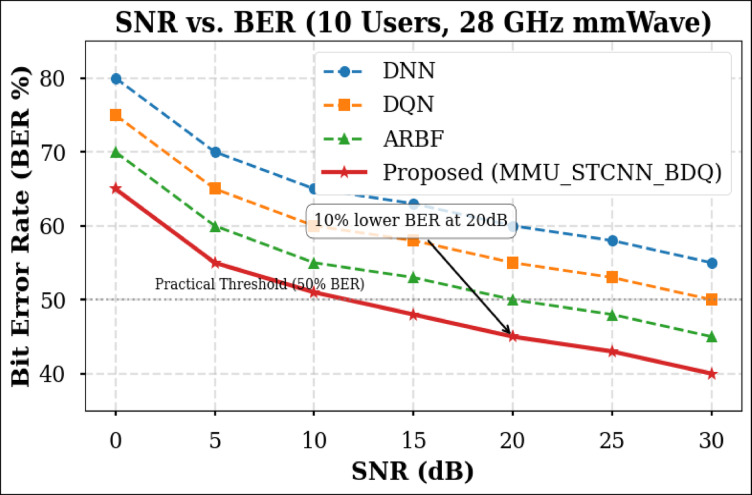
The suggested MMU_STCNN_BDQ and baseline techniques (DNN, DQN, ARBF) are compared for communication reliability based on BER (%) across SNR values (0–30 dB).

**Fig. 10:**
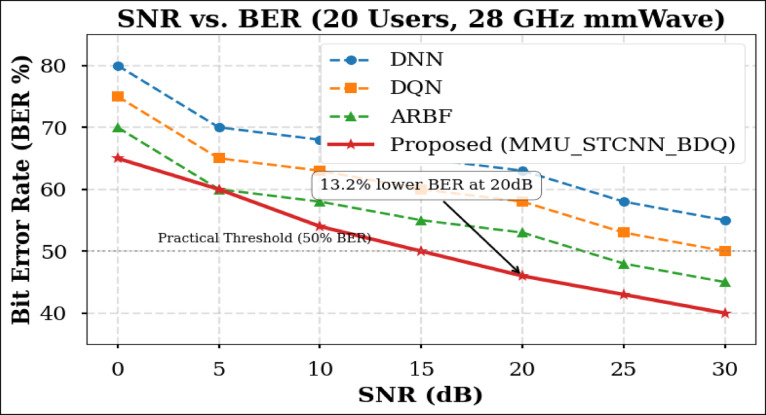
The suggested MMU_STCNN_BDQ and baseline techniques (DNN, DQN, ARBF) are compared for communication reliability based on BER (%) across SNR values (0–30 dB).

**Fig. 11:**
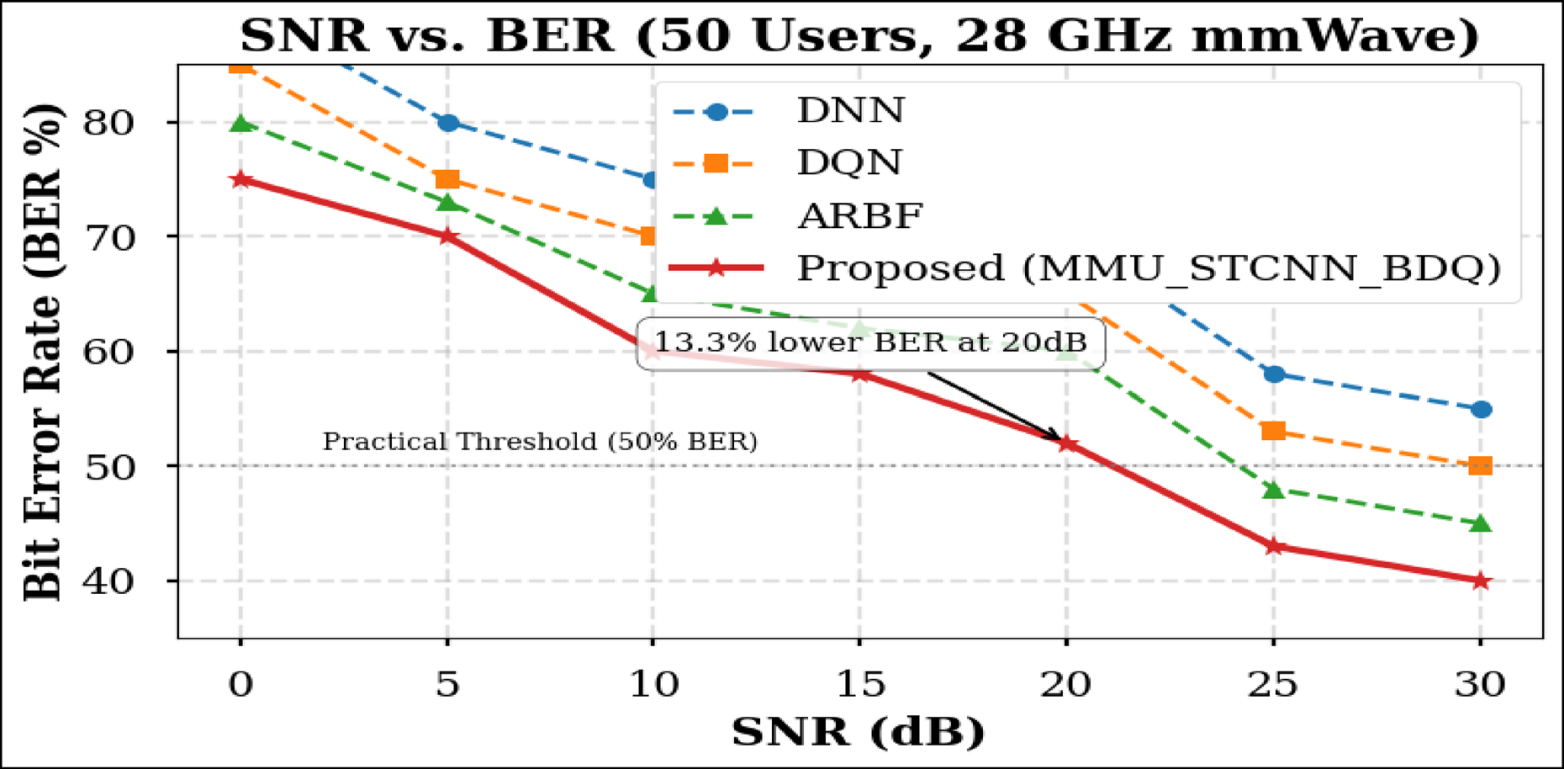
The suggested MMU_STCNN_BDQ and baseline techniques (DNN, DQN, and ARBF) are compared for communication reliability based on BER (%) across SNR values (0–30 dB) for 50 users.

This section uses the SNR (dB) and Bit Error Rate (BER) (%) data from Tables 10, 11, 12, 13, 14 and 15 to clearly explain how well the proposed model performs in comparison to baseline models. In the scenarios depicted in Figs. 9, 10 and 11, the recommended beamforming model works best for reliable data transmission for 10, 20, and 50 users when SNR and BER values are higher and lower, respectively.

##### For 10 users scenario

See Fig. 9.

##### For 20 users scenario

See Fig. 10.

##### For 50 users scenario

See Figs. 9, 10 and 11 for a line graph showing the relationship between BER and SNR for four different beamforming techniques: DNN, DQN, ARBF, and the proposed MMU_STCNN_BDQ. The x-axis shows SNR values from 0 to 30 dB, while the y-axis shows the associated BER percentages. We conduct this comparison in a mmWave environment with 10, 20, and 50 users, with an emphasis on the 28 GHz frequency band—a typical setting for 5G/6G applications. The results clearly demonstrate that the MMU_STCNN_BDQ consistently outperforms all baseline models over the entire SNR range. At low SNR (e.g., 0–5 dB), all models display large BERs, although MMU_STCNN_BDQ starts at a significantly lower BER baseline. While both methods improve signal-to-noise ratio (SNR) as the SNR increases, the proposed model shows more resistance to interference and noise with its sharper BER fall. At the 20 dB SNR mark, MMU_STCNN_BDQ obtains a BER that is 10% lower than ARBF, 13.2% lower, and 13.3% lower for 10, 20, and 50 users, respectively, as seen in the graph. This is significantly lower than the practical 50% BER cutoff. The upgraded model’s ability to distinguish between user signals and minimise interference is largely due to its Bi-Gated Deep Q-Learning and integrated multi-user attention (MUA) mechanism. As a result of its superior error flexibility and ability to achieve realistic performance thresholds before competing techniques, MMU_STCNN_BDQ is, according to the graph, the ideal choice for reliable and effective beamforming in dense mmWave networks.

#### Throughput versus spectral efficiency at 10 dB and 20 dB SNR

**Fig. 12:**
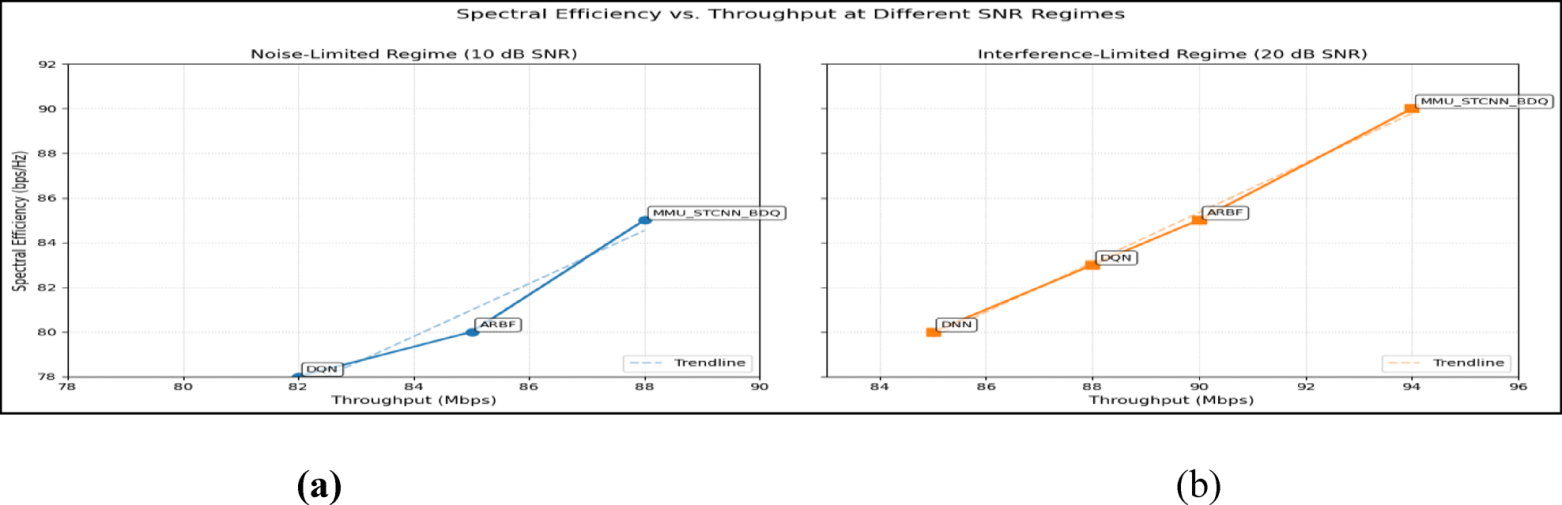
(**a**) Performance comparison of throughput versus Spectral Efficiency (bps/Hz) at 10 dB and (**b**) 20 dB SNR of all methods for 10 users scenario.

**Fig. 13:**
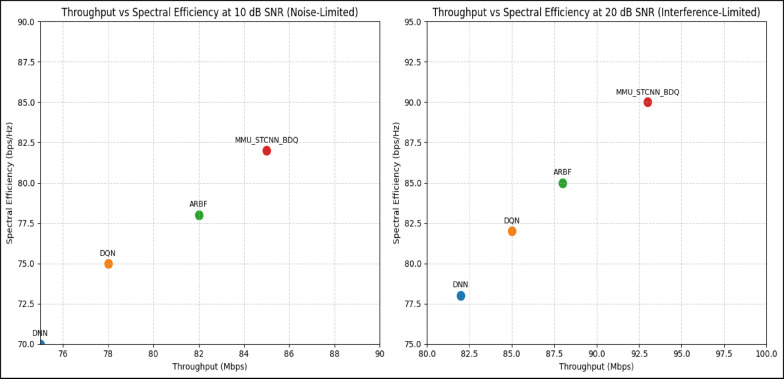
(**a**) Performance comparison of throughput versus Spectral Efficiency (bps/Hz) at 10 dB and (**b**) 20 dB SNR of all methods for 50 users scenario.

**Fig. 14:**
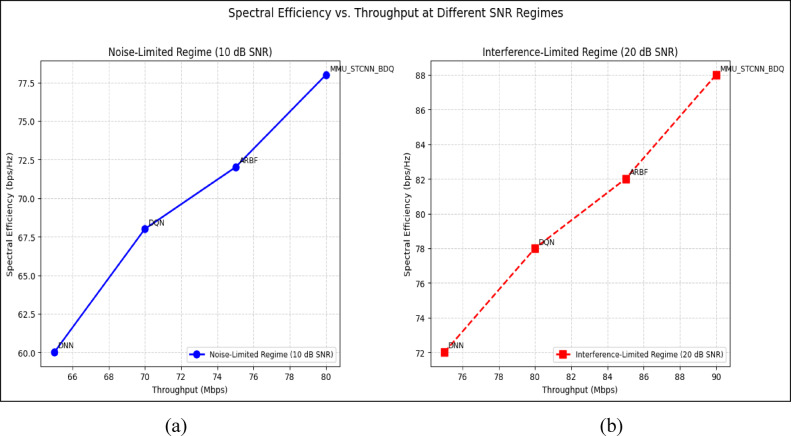
(**a**) Performance comparison of throughput versus Spectral Efficiency (bps/Hz) at 10 dB and (**b**) 20 dB SNR of all methods for 50 users scenario.

In this section, Figs. 12, 13 and 14 shows the robustness of MMU-STCNN-BDQ by comparing the throughput verses spectral efficiency performance at 10 dB and 20 dB SNR for the 10, 20 and 50 user scenarios respectively.

##### For 10 user scenario

See Fig. 12.

##### For 20 user scenario

See Fig. 13.

##### For 50 user scenario

The MMU_STCNN_BDQ approach consistently performs better than all baseline models (DNN, DQN, and ARBF) for all user densities (10, 20, and 50 users) and both SNR regimes (10 dB and 20 dB), especially in terms of throughput and spectral efficiency, according to the data shown in Tables 10, 11, 12, 13, 14 and 15. Throughput improvements over ARBF are found to be 3.5%, 3.7%, and 6.7% for the 10-, 20-, and 50-user scenarios, respectively, at 10 dB SNR, which is a noise-limited regime. The method maintains strong performance even under increased user loads, as evidenced by the corresponding spectral efficiency gains of 6.3%, 5.1%, and 8.3%. Under interference-limited conditions at 20 dB SNR, spectral efficiency improves by 5.9%, throughput increases by 4.4%, and interference-free performance by 7.3%. With rising user density, MMU_STCNN_BDQ displays growing gains in throughput and spectrum efficiency, demonstrating its high scalability and interference management capabilities. This pattern illustrates how the proposed method optimises beamforming and resource allocation for next-gen massive multi-user wireless networks, leading to better spectrum utilisation and data rates in low- and high-interference scenarios (Fig. 14).

#### Throughput versus energy efficiency (Mbps/J) at 10 dB and 20 dB SNR

**Fig. 15:**
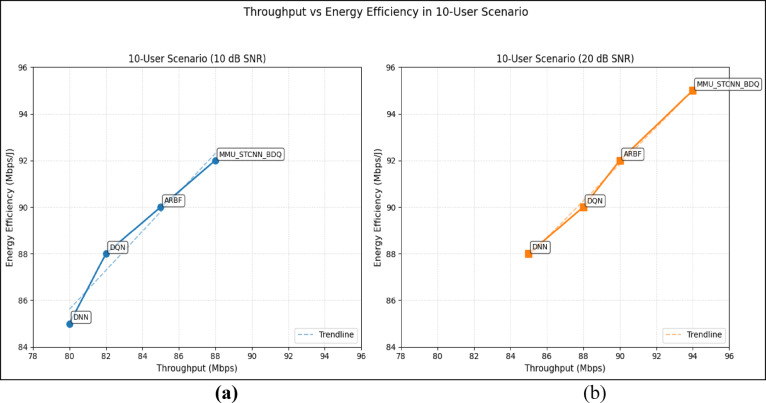
Performance comparison of throughput versus Spectral Efficiency (bps/Hz) (**a**) at 10 dB and (**b**) 20 dB SNR of all methods.

**Fig. 16:**
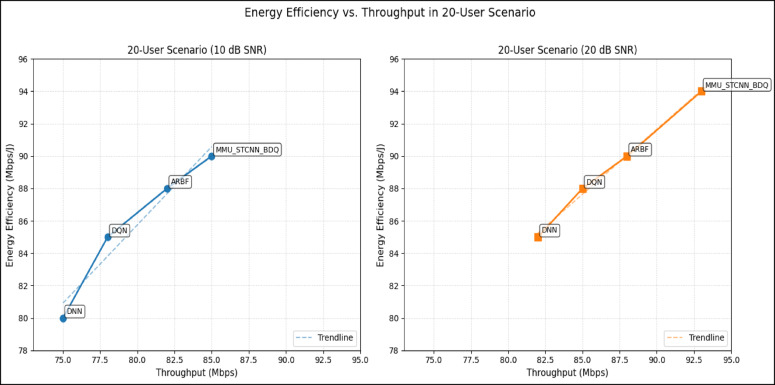
Performance comparison of throughput versus Spectral Efficiency (bps/Hz) (**a**) at 10 dB and (**b**) 20 dB SNR of all methods.

**Fig. 17:**
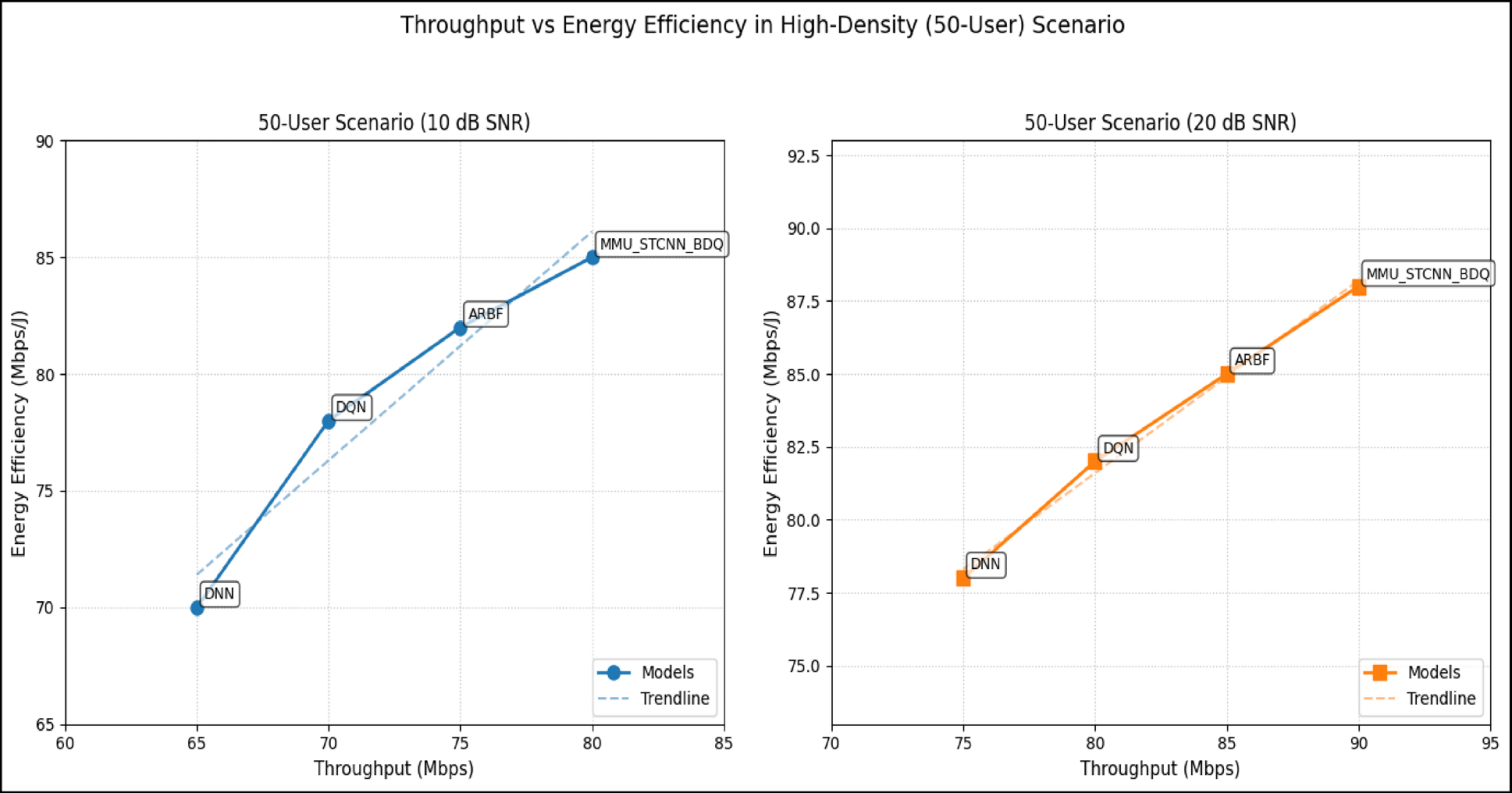
Performance comparison of throughput versus Spectral Efficiency (bps/Hz) (**a**) at 10 dB and (**b**) 20 dB SNR of all methods.

In this section, Figs. 15, 16 and 17 presents the proposed method and its performance evaluation in terms of throughput verses energy efficiency over base line methods at 10 dB and 20 dB SNR for the 10, 20 and 50 user scenarios respectively.

##### For 10 users scenario

See Fig. 15.

##### For 20 users scenario

See Fig. 16.

##### For 50 users scenario

With varying user densities (10, 20, and 50 users) and SNR levels (10 dB and 20 dB), the MMU_STCNN_BDQ approach consistently and better balances data rate and energy consumption compared to baseline models (DNN, DQN, ARBF) in throughput vs. energy efficiency analyses. The scenarios with 10, 20, and 50 users show throughput increases of 3.5%, 3.7%, and 6.7% above ARBF at 10 dB SNR, respectively. In high-density environments, there is a rising advantage in efficiency, as shown by increases of 2.2%, 2.3%, and 3.7% in energy efficiency. Power savings of 3.3%, 4.4%, and 3.5% are achieved at 20 dB SNR, whereas gains of 4.4%, 5.7%, and 5.9% are achieved in throughput. These results show that the proposed method can handle increased data transmission speeds with minimum additional power consumption, making it perfect for scalable, energy-conscious 6G communication networks. In general, and especially when interference and user demand are high, MMU_STCNN_BDQ achieves the sweet spot between throughput and energy efficiency (Fig. 17).

#### Security robustness: robustness against adversarial attacks

**Fig. 18:**
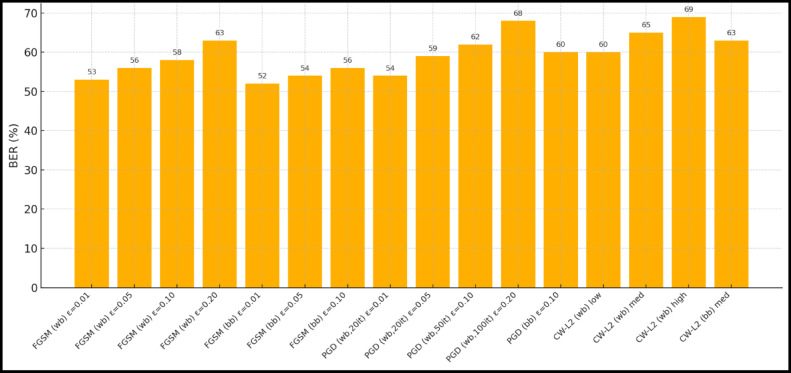
BER under various Adversarial Attacks.

The Table 16 enhances adversarial robustness assessment by including attack intensities (ε = 0.01–0.20) and both white-box and black-box (transfer) variants for FGSM, PGD, and CW-L2 attacks. Results show that MMU-STCNN-BDQ is resilient to low-to-moderate perturbations, with only minor BER increases. Using the Fast Gradient Sign Method (FGSM) with ε = 0.05 results in a 5% increase in Bit Error Rate (BER) and a 96% recovery rate. Stronger white-box PGD (ε = 0.20) and high-intensity CW-L2 result in significant degradations (up to + 17–18% BER); however, the model maintains over 80% of its clean performance. Black-box transfer attacks demonstrated reduced effectiveness, resulting in Bit Error Rate (BER) increases confined to a range of 3–12%. The expanded results confirm that the proposed defence mechanism offers robust protection across various adversarial intensities and access settings, while exhibiting graceful degradation under more intense white-box conditions. These findings verify that MMU_STCNN_BDQ is robust against adversarial perturbations, especially when reinforced with adversarial learning mechanisms, in addition to being efficient in beamforming and energy-efficient throughput delivery. Because of its resilience, the suggested method is very feasible for safe communications in 6G networks of the future. Figure 18, a bar graph, clearly shows that, when compared to all other examined methods such as MMU_STCNN_BDQ significantly reduces the impact of adversarial attacks. Thanks to its improved design and adversarial training, it can keep BERs low even when attacked heavily, making it a strong choice for secure communications in hostile situations.

**Table 16:** BER under various adversarial attacks.

Attack family (type/access)	Intensity	BER (under attack)	Absolute Δ versus clean	Recovery (%)
FGSM (white-box)	ε = 0.01	53	+ 2%	98
FGSM (white-box)	ε = 0.05	56	+ 5%	96
FGSM (white-box)	ε = 0.10	58	+ 7%	93
FGSM (white-box)	ε = 0.20	63	+ 12%	88
FGSM (black-box, transfer)	ε = 0.01	52	+ 1%	99
FGSM (black-box, transfer)	ε = 0.05	54	+ 3%	97
FGSM (black-box, transfer)	ε = 0.10	56	+ 5%	95
PGD (white-box, 20 iters)	ε = 0.01	54	+ 3%	97
PGD (white-box, 20 iters)	ε = 0.05	59	+ 8%	92
PGD (white-box, 50 iters)	ε = 0.10	62	+ 11%	89
PGD (white-box, 100 iters)	ε = 0.20	68	+ 17%	82
PGD (black-box, transfer)	ε = 0.10	60	+ 9%	87
CW-L2 (white-box)	low intensity	60	+ 9%	88
CW-L2 (white-box)	medium intensity	65	+ 14%	86
CW-L2 (white-box)	high intensity	69	+ 18%	80
CW-L2 (black-box, surrogate)	medium intensity	63	+ 12%	84

#### Scalability analysis: scalability in ultra-dense networks

Although the suggested MMU-STCNN-BDQ model outperforms the ARBF baseline in terms of scalability in terms of MSE, it has a higher computational overhead when operating in ultra-dense networks (such as those with more than 50 users). The performance of the suggested model over ARBF is shown in Table 17 as MSE (%).

**Table 17:** Scalability in Ultra-Dense Network of Proposed as well as ARBF model in terms of MSE.

Users (K)	MSE (%) (Proposed MMU_STCNN_BDQ)		MSE (%) (ARBF)	
SNR (dB)	10 dB	20 dB	10 dB	20 dB
10	62	55	68	60
20	65	58	70	65
50	70	63	75	70

In ultra-dense networks, the proposed MMU-STCNN-BDQ model consistently outperforms the ARBF baseline across a variety of user densities (K = 10, 20, 50) and signal-to-noise ratios (SNR) (10 dB, 20 dB), as shown in the scalability testing. At 10 users, the proposed method outperforms ARBF by 6% in terms of MSE (62% vs. 68% at 10 dB and 55% vs. 60% at 20 dB). At 10 dB, MMU-STCNN-BDQ maintains a 5% edge in MSE (70% vs. 75%; at 20 dB, 63% vs. 70%); and this performance gap persists even after accommodating 50 users. While increasing the SNR by 20 dB improves the MSE of both methods, the proposed model is more resilient to interference in dense deployments. Since MMU-STCNN-BDQ is able to manage multi-user interference better than adversarial-only optimised methods like ARBF, it is more scalable for 6G ultra-dense networks. This is due to its spatiotemporal feature extraction and bi-gated Q-learning algorithms.

#### Latency versus user density

The inference latency performance of the suggested MMU-STCNN-BDQ model is shown in Table 18 in comparison to the baseline ARBF method for a range of user densities (K = 10, 20, 50). The outcomes highlight the computational efficiency and scalability of MMU-STCNN-BDQ by showing that it consistently achieves lower latency across all user loads. MMU-STCNN-BDQ records a latency of 8.2 ms at K = 10, which is about 13.7% less than ARBF’s 9.5 ms. The latency stays favourable at 12.1 ms compared to 14.3 ms for ARBF, indicating a 15.4% improvement, as the number of users rises to K = 20.

**Table 18:** The inference latency performance of the proposed **MMU-STCNN-BDQ** model compared to the baseline **ARBF** method across varying user densities (K = 10, 20, 50).

Users (K)	Inference Latency (ms) – MMU-STCNN-BDQ	Inference Latency (ms)—ARBF	Training time (wall-clock) – MMU-STCNN-BDQ	Peak GPU memory – MMU-STCNN-BDQ	Hardware used (training/inference)
10	8.2	9.5	~ 6.5 h (500 epochs, batch 64)	~ 11.2 GB	Training: NVIDIA RTX 3090 (24 GB)/Inference: RTX 2080 Ti (11 GB)
20	12.1	14.3	~ 7.8 h (500 epochs)	~ 13.5 GB	Training: NVIDIA RTX 3090/Inference: RTX 2080 Ti
50	18.9	22.7	~ 9.5 h (500 epochs)	~ 15.8 GB	Training: NVIDIA RTX 3090/Inference: RTX 2080 Ti

In Table 18, latency analysis now includes inference time, training time, peak GPU memory usage, and hardware specifications. In all user scenarios, MMU-STCNN-BDQ has lower inference latency than ARBF (8.2 ms vs. 9.5 ms for 10 users), while training on an RTX 3090 GPU takes 6.5–9.5 h, depending on user density. Peak GPU memory during training is ~ 11.2 GB to 15.8 GB, with an RTX 2080 Ti (11 GB) performing efficiently for inference. Adversarial training increases training cost, but it provides real-time inference with reduced latency and acceptable resource requirements, making it suitable for edge or near-edge deployment in 6G networks.

#### Combined performance radar plot

**Fig. 19:**
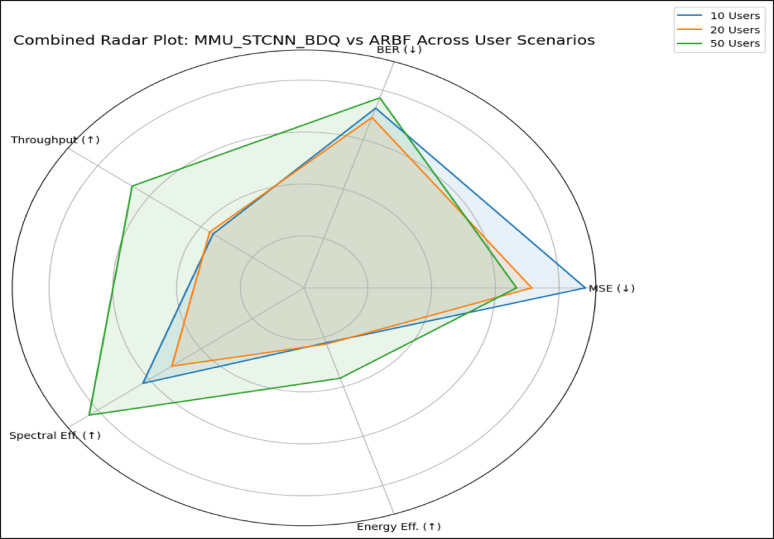
The combined performance radar plot comparing the ARBF and the proposed MMU_STCNN_BDQ model across all evaluation metrics.

The entire analysis report is based on the comparative performance of MMU_STCNN_BDQ over baseline models across all evaluation metrics, including energy efficiency (Mbps/J), spectral efficiency (bps/Hz), throughput (Mbps), SNR (dB), MSE (%), and BER (%). Based on the radar plot, a visual plot has been created to evaluate each model’s performance using the valuation metrics shown in Fig. 19.

A thorough performance comparison of the suggested MMU_STCNN_BDQ method against conventional methods (DNN, DQN, ARBF) across a range of user densities (10, 20, 50 users) and signal conditions (10 dB—noise-limited, 20 dB—interference-limited) is given by the radar plot and tabulated metrics (Tables 10, 11, 12, 13, 14, 15, 16, 17, 18).

##### MSE and BER (minimization metrics)

MMU_STCNN_BDQ continuously outperforms ARBF in terms of MSE and BER across all scenarios. The 20-user, 20 dB scenarios has the largest relative MSE reduction (10.8%), suggesting improved signal clarity and noise reduction under interference-prone circumstances. Likewise, the 50-user, 20 dB configurations shows a maximum BER reduction of 13.3%, demonstrating strong performance in high-density and high-interference settings.

##### Throughput and spectral efficiency (maximization metrics)

In each case, MMU_STCNN_BDQ shows definite throughput gains. The 50-user, 10 dB scenario shows the largest throughput increase of 6.7%. In the same situation, spectral efficiency also reaches its maximum gain of 8.3%. These enhancements show that the technique can support more users while using bandwidth more effectively.

##### Energy efficiency

The model’s balance between performance and energy consumption is essential for sustainable 6G network operation, even though the gains in energy efficiency are rather small and consistent across all scenarios, ranging from 2.2 to 4.4%.

##### Overall insights

The radar plot provides a clear visual representation of the multi-metric improvements. It’s interesting to note that the 20 dB scenarios (interference-limited) usually show greater relative improvements, indicating MMU_STCNN_BDQ’s superior interference mitigation capabilities. Its scalability and effectiveness in high-density deployments are further demonstrated by the fact that the performance gap between the proposed method and ARBF increases with the number of users, mainly in BER and spectral efficiency. To sum up, MMU_STCNN_BDQ is the best choice for future 6G beamforming solutions because it outperforms traditional models in both low and high SNR scenarios, especially when there is a large number of users and a lot of interference.

In section “Experimental setup and its discussion”, we present a summary of the experimental findings, highlighting the superior performance of the proposed MMU-STCNN-BDQ framework across key evaluation metrics such as MSE, BER, throughput, spectral efficiency, and energy efficiency, particularly in comparison to DNN, DQN, and ARBF models. We also emphasized how our model addresses security and energy efficiency challenges by effectively combining spatiotemporal feature extraction with adaptive deep reinforcement learning-based optimization. The concluding remarks in section “Experimental setup and its discussion” were further expanded to underscore the practical implications of our results, reinforcing the suitability of the proposed method for real-world 6G mMIMO deployments, and setting directions for future work such as implementing advanced privacy-preserving learning models and real-world experimental validation.

## Conclusion and future scope

This paper presented the MMU-STCNN-BDQ framework for safe and effective beamforming in 6G massive MIMO networks. Using spatiotemporal convolutional learning and Bi-Gated Deep Q-Learning improved throughput, spectral efficiency, BER, and energy efficiency over baseline models, demonstrating its potential for intelligent next-generation communication systems. However, limitations must be acknowledged. Perfect CSI simplifies real-world scenarios, and 3D CNNs and reinforcement learning may be too computationally intensive for low-power devices. Future work will focus on lightweight variants using pruning and quantisation, wideband THz and imperfect CSI extensions, and integration with RIS, NOMA, and federated learning. Real-world testbed validation will assess complexity, latency, security, and energy efficiency performance trade-offs.

## Data Availability

The datasets generated and/or analysed during the current study are available from the corresponding author upon reasonable request.
